# Bayesian spatial modelling of childhood cancer incidence in Switzerland using exact point data: a nationwide study during 1985–2015

**DOI:** 10.1186/s12942-020-00211-7

**Published:** 2020-04-17

**Authors:** Garyfallos Konstantinoudis, Dominic Schuhmacher, Roland A. Ammann, Tamara Diesch, Claudia E. Kuehni, Ben D. Spycher, for the Swiss Paediatric Oncology Group, R. A. Ammann, K. Scheinemann, M. Ansari, M. Beck Popovic, P. Brazzola, J. Greiner, M. Grotzer, H. Hengartner, T. Kuehne, J. Rössler, F. Niggli, F. Schilling, N. von der Weid, the Swiss National Cohort Study Group, Matthias Egger, Adrian Spoerri, Marcel Zwahlen, Milo Puhan, Matthias Bopp, Martin Röösli, Murielle Bochud, Michel Oris

**Affiliations:** 1 Institute of Social and Preventive Medicine (ISPM), University of Bern, Bern, Switzerland; 2 Epidemiology and Biostatistics Department, School of Public Health, Imperial College London, London, UK; 3 Institute for Mathematical Stochastics, University of Göttingen, Göttingen, Germany; 4 Department of Paediatrics Inselspital, Bern University Hospital, University of Bern, Bern, Switzerland; 5 Division of Paediatric Oncology/Haematology, University Children’s Hospital Basel, Basel, Switzerland

**Keywords:** Cancer clusters, Central nervous system cancer, Childhood cancer, Bayesian spatial modelling, Point processes

## Abstract

**Background:**

The aetiology of most childhood cancers is largely unknown. Spatially varying environmental factors such as traffic-related air pollution, background radiation and agricultural pesticides might contribute to the development of childhood cancer. This study is the first investigation of the spatial disease mapping of childhood cancers using exact geocodes of place of residence.

**Methods:**

We included 5947 children diagnosed with cancer in Switzerland during 1985–2015 at 0–15 years of age from the Swiss Childhood Cancer Registry. We modelled cancer risk using log-Gaussian Cox processes and indirect standardisation to adjust for age and year of diagnosis. We examined whether the spatial variation of risk can be explained by modelled ambient air concentration of NO_2_, modelled exposure to background ionising radiation, area-based socio-economic position (SEP), linguistic region, duration in years of general cancer registration in the canton or degree of urbanisation.

**Results:**

For all childhood cancers combined, the posterior median relative risk (RR), compared to the national level, varied by location from 0.83 to 1.13 (min to max). Corresponding ranges were 0.96 to 1.09 for leukaemia, 0.90 to 1.13 for lymphoma, and 0.82 to 1.23 for central nervous system (CNS) tumours. The covariates considered explained 72% of the observed spatial variation for all cancers, 81% for leukaemia, 82% for lymphoma and 64% for CNS tumours. There was weak evidence of an association of CNS tumour incidence with modelled exposure to background ionising radiation (RR per SD difference 1.17; 0.98–1.40) and with SEP (1.6; 1.00–1.13).

**Conclusion:**

Of the investigated diagnostic groups, childhood CNS tumours showed the largest spatial variation. The selected covariates only partially explained the observed variation of CNS tumours suggesting that other environmental factors also play a role.

## Background

The causes of childhood cancers are poorly understood. Epidemiological research on the atomic bomb survivors indicated that ionising radiation in high doses can cause childhood leukaemia and central nervous system (CNS) tumours [1, 2]. A number of environmental factors have been suggested that could partially explain cancer risks in the general population, including traffic-related air pollution [3], background radiation [2, 4] and agricultural pesticides [5]. These risk factors vary in space and it is thus natural to expect spatial variation in childhood cancer incidence. Conversely, investigating the spatial variation of childhood cancer incidence might help generate new hypotheses about environmental risk and identify areas of potential environmental contamination.

Disease mapping is a common way of capturing the spatial variation of a disease. Direct estimation of disease incidence in small areas is subject to large sampling variability, particularly for rare diseases. Disease mapping mitigates this problem by exploiting spatial correlation between neighbouring areas and smoothing small area rates based on neighbouring values [6]. Several previous studies have investigated spatial variation in childhood cancer risk using disease mapping. Studies focusing on childhood leukaemia reported evidence of spatial variation in Ohio, USA [7], Texas, USA [8], Yorkshire, UK [9], but not in France [10]. The study in Texas also examined childhood lymphomas and reported some evidence of spatial variation of Hodgkin lymphoma [8]. A study in Kenya reported evidence of spatial variation of Burkitt’s lymphoma with higher rates in the northern part of the country [11]. A study in Florida, USA, focusing on childhood brain tumours reported some evidence of high excess risk in several non-adjacent counties [12].

The different findings regarding spatial variation of childhood cancers might reflect differences between the countries or methodological limitations. Most previous studies relied on areal data (data aggregated on administrative units) [8–10, 12–14]. Results from such studies depend on spatial unit selected, which is referred to as the Modifiable Areal Unit Problem [15]. Furthermore, associations between cancer incidence and environmental factors assessed at group level may be subject to ecological fallacy, i.e. they may not correctly reflect underlying associations at the individual level [16]. In a simulation study, we showed that spatial modelling based on exact geocodes is more accurately identifies areas of higher risk compared to traditional disease mapping based on count data aggregated to small administrative areas [17]. To the best of our knowledge, only one previous study used precise geocodes, but the authors did not attempt to explain the observed variation of childhood leukaemia risk by incorporating environmental exposures in the model [7]. Lastly, all the previous studies used geographical information about the place of diagnosis of the cancer cases. Children may be more susceptible to certain environmental exposures early in life and thus location of residence at birth may be more relevant [18].

In this nationwide study, we investigated the spatial variation of childhood cancers in Switzerland using precise locations of residence. We performed analysis using place of birth and diagnosis. We focused on the following main diagnostic groups: all childhood cancers, childhood leukaemia, lymphoma and CNS tumours and assessed the extent to which selected covariates could explain the observed spatial variation.

## Methods

### Study population

We retrieved children diagnosed with cancer in Switzerland during 1985–2015 at age 0–15 from the Swiss Childhood Cancer Registry (SCCR). SCCR is a nationwide registry with high completeness. Estimates suggest that it includes 91% of all incident cases for the period 1985–2009 and > 95% for 1995–2009 [19]. It collects residential addresses from time of diagnosis back to birth. The addresses were geocoded according to the Swiss grid coordinate system using a combination of different sources of georeferenced building addresses including the Swiss postal system, the geoportal maintained by the Federal Office of Topography (swisstopo; http://map.geo.admin.ch), and Google Maps. We wrote an R function to assign a pair of geocodes to every address (https://github.com/gkonstantinoudis/GeoSwiss) based on these data sources. Geocodes that could not be identified by this procedure where manually searched using the mentioned web sites. If the available address information was incomplete (e.g. only street without number, or only postal code without street), approximated geocodes were assigned (e.g. a central location on the street, or of the municipality). We classified cases based on the margin of error 1 (< 50 m), 2 (> 50 m and < 100 m), 3 (> 100 m and < 500 m) and 4 (> 500 m). For 94% of the cases, we geocoded residential addresses with a margin of error < 100 m.

Population data was available through the Swiss National Cohort (SNC) which includes exact geocoded residential locations of all Swiss residents at the times of censuses (1990, 2000 and 2010–2015). To calculate population at risk by age group, year and spatial unit (1 km^2^ grid cell or municipality), we performed linear interpolation of age, year and spatial unit specific weights, see Additional file 1: Text S1 and Figures S1, S2. The 1 km^2^ grid size was selected as a compromise between our goal of high precision maps on the one hand, and data confidentiality considerations and reduction of computational burden on the other. We then performed indirect standardisation by calculating the expected number of cases adjusted by age and year: Let $q_{i,j}$ be the nationwide cancer incidence rate and $P_{i,j,k}$ the population counts with subscript referring to the $i$-th age group (0–4, 5–9, 10–15), $j$-th year (1985–2015), and $k$-th spatial unit (grid cell, or municipality). Then the expected number of cases in the $k$-th spatial unit is:

$$E_{k} = \mathop \sum \limits_{i} \mathop \sum \limits_{j} q_{i,j} \cdot P_{i,j,k} .$$

To calculate the expected number of cases for the analysis based on the location at birth we used a similar procedure restricting to children aged < 1 year at census:

$$E_{k} = \mathop \sum \limits_{j} q_{0,j} \cdot P_{0,j,k} ,$$

where the age index i = 0 represents children aged < 1 year. We repeated the aforementioned procedures for the different diagnostic groups. For more information refer to Additional file 1: Text S1.

### Outcomes

The SCCR classifies diagnoses according to the International Classification of Childhood Cancers Third Edition (ICCC3). We examined all childhood cancers combined (ICCC3 main groups I–XII) and then separately childhood leukaemia (ICCC3 main group I), lymphoma (ICCC3 main group II) and CNS tumours (ICCC3 main group III). We focused on the main diagnostic groups because of the larger sample size.

### Covariates

As potential explanatory variables, we included predicted ambient air concentration of NO_2_, predicted total dose rate from terrestrial gamma and cosmic radiation, neighbourhood-level socio-economic position (Swiss-SEP for the year 2001) [20], years of general cancer registration in the canton, language region and the degree of urbanisation as covariates (Additional file 1: Table S1 and Figures S3–S8). Traffic-related air pollution and total background radiation were previously found to be associated with childhood cancer risks in Switzerland [21, 22]. We included SEP, linguistic region and degree of urbanisation to account for regional, socio-economic and socio-cultural differences. We included years of cantonal cancer registration to account for heterogeneous registry completeness. The SCCR records childhood cancer cases treated in one of the nine specialised paediatric oncology (SPOG) clinics and complements the registry with any additional cases recorded by the cantonal registries. Some cantons already had a cancer registry at the beginning of our study period, others established one during the study period and others after the end of the study. For cantons with more years of general registration, we thus expect the “apparent” childhood cancer incidence over the study period to be slightly higher.

### Statistical analysis

We used log-Gaussian Cox processes (LGCPs) to model locations of incident cancer cases [23]. The point process assumed to generate the case locations is an inhomogeneous Poisson process with random intensity $e\left( s \right)r\left( s \right)$, $s \in W$, where $e\left( s \right)$ denotes the intensity of the expected number of cases (approximated with $E_{k}$), $r\left( s \right)$ the risk and $W$ denotes the observation window (Switzerland in our case). We model the continuous log-risk surface $\log r\left( s \right), s \in W$, via a spatial mixed effects model $\varvec{X}\left( s \right)\varvec{\beta}+ Z\left( s \right)$, where the first summand adjusts for covariate effects and the second summand models the spatial variation. The process $Z\left( s \right)$ is assumed to be a zero mean Gaussian random field (GRF) with Matérn covariance function. We fix the smoothness parameter $\nu$ to 1, which is common practice to alleviate the computational burden [24]. The Gaussian field is then controlled by two parameters: a variance parameter $\sigma^{2}$ and a range parameter $\rho$(roughly, the distance between $s$ and $t$ in $W$ at which the correlation between the values $Z\left( s \right)$ and $Z\left( t \right)$ of the field falls below 0.10). To be avoid large dense covariance matrices, we use the approach by Lindgren et al. [25]. Thus, we approximate the field $Z\left( s \right)$, $s \in W$, by a finite element representation of the (weak) solution of a certain stochastic partial differential equation (SPDE):

$$Z\left( s \right) \approx \mathop \sum \limits_{i = 1}^{M} \psi_{i} \left( s \right)Z_{i} = :Z_{*} \left( s \right),$$

where $M$ denotes the total number of nodes in an underlying triangulation of $W$, $\psi_{i}$ are piecewise linear basis functions taking the value 1 at the $i$-th node and 0 at every other node, and $Z_{i}$ are random weights forming a (finitely indexed) Gaussian Markov random field (GMRF) $\varvec{Z} = \left( {Z_{i} } \right)_{i = 1, \ldots ,M}$. The latter has a sparse precision matrix $\varvec{Q}\left( {\rho , \sigma } \right)$. We used penalised complexity priors (PC priors) for the hyperparameters $\sigma$ and $\rho$. For the construction of these priors in the present GRF setting, see [26]. The full model specification then reads:

$$\log r\left( s \right) = \varvec{X}\left( s \right)\varvec{\beta}+ Z_{*} \left( s \right)$$

$$\varvec{Z} \sim N\left( {0, \varvec{Q}\left( {\rho , \sigma } \right)^{ - 1} } \right)$$

$$\varvec{\beta}\sim N\left( {0, 10\varvec{I}} \right)$$

$$\sigma \sim PCprior\left( {0.01, 1} \right)$$

$$\rho \sim PCprior\left( {0.5, 60} \right)$$

where $\varvec{X}\left( s \right)$ = $\left( {X_{0} \left( s \right), X_{1} \left( s \right), \ldots , X_{l} \left( s \right)} \right)$ is a row vector of spatial covariates and $\varvec{I}$ is the identity matrix. The user-defined scale of the PC prior of the standard deviation $\sigma$ was chosen such that the variance of the log relative risk at any fixed location exceeds 1 with probability 0.01. The scale for the range parameter $\rho$ was adjusted so that the probability of having range smaller than 60 km is 0.50. We used the Integrated Nested Laplace Approximation (INLA) to render Bayesian analysis for the above model computationally feasible [27, 28].

We computed maps of posterior median (unadjusted or adjusted for the covariates) of spatial relative risk (RR, i.e. ${\text{exp}}\left\{ {Z\left( s \right)} \right\}$) compared to national level on a $1 \times 1\;{\text{km}}^{2}$ grid. We also mapped exceedance probabilities defined as the posterior probability, in each grid cell, that RR exceeds 1. The fixed effects $\beta_{i}$ (log-relative-risk per unit increase in the covariate) are reported as posterior median of RR, i.e. ${ \exp }\left\{ {\beta_{i} } \right\}$, and 95% credibility intervals (CI). The continuous variables NO_2_, ionizing radiation, SEP and years of cantonal cancer registration were scaled and thus ${ \exp }\left\{ {\beta_{i} } \right\}$ is interpreted as the multiplicative change of the risk at a fixed location when the covariate is increased by 1 standard deviation (SD). They were included as linear terms since there was no indication for a more complex model (Additional file 1: Figure S9). Henceforth, the model adjusted for the aforementioned covariates is referred to as the adjusted model, whereas the model without covariates as the unadjusted. Both adjusted and unadjusted models were standardised for population, age and year of diagnosis by including the expected number of cases as an offset in the model (Additional file 1: Text S1, S2).

We also report the percentage of variance explained by the selected risk factors by evaluating median and 95% CI of the posterior of an extension of Bayesian $R^{2}$ [29]:

$$R^{2} = \frac{{V\left( {\varvec{X}\left( s \right)\varvec{\beta}} \right)}}{{V\left( {\varvec{X}\left( s \right)\varvec{\beta}} \right) + V\left( {Z\left( s \right)} \right)}},$$

where $V\left( \cdot \right)$ denotes the variance over the $K$ spatial units, $\varvec{\beta}$ is the vector of intercept and covariates and $\varvec{X}\left( s \right)$ is the design matrix. We calculated $R^{2}$ for the fully adjusted model, a model including all selected covariates except years of cantonal cancer registration (we refer to this set of covariates as ‘putative risk factors’), and the univariable model including only years of cantonal cancer registration. This allowed us to distinguish spatial variation explained purely by the degree of completeness of registration from variation explained by covariates that might reflect aetiological factors (putative risk factors). For consistency with the literature, we also fitted the Besag-York-Mollié (BYM) model using disease counts per municipality, for more information see [26, 30, 31] and Additional file 1: Text S2

### Sensitivity analysis

We ran a sensitivity analysis to examine the robustness of the results with respect to different scalings of the penalized complexity priors for the range parameter of the latent field [26], with median range fixed at $1, 10, 60, 120$ and $240\;\;{\text{km}}$.

## Results

### Study population

We identified 5969 cases with childhood cancer during 1985–2015 in Switzerland. We excluded 22 (0.3%) cases without available geocode of residence at diagnosis. Of the included 5947 children, 32% (N = 1880) had leukemia, 13% (N = 772) lymphoma and 22% (N = 1290) a CNS tumor. For the analysis using location at birth we first excluded 1194 cases born before 1985 and then 577 additional cases with no geocode at birth yielding 4198 cases for the analysis (Table 1). Of the excluded cases for this analysis, 342 were born abroad, 114 were born in Switzerland but no address was recorded, while for 121 the country of birth was missing. The age and sex distribution follows similar patterns as in neighbouring countries (Table 1) [32, 33].

**Table 1:** Number of cases and median age at diagnosis for the analysis based on the location at birth and diagnosis

	Birth			Diagnosis		
	Total N (%)	Female N (%)	Median age at diagnosis	Total N (%)	Female N (%)	Median age at diagnosis
All cancers	4198 (100)	1875 (45)	4.8	5947 (100)	2654 (45)	6.4
Leukaemia	1384 (33)	570 (41)	4.2	1880 (32)	781 (42)	4.9
Lymphoma	459 (11)	161 (35)	10.2	772 (13)	279 (36)	11.5
CNS tumours	902 (21)	421 (47)	6.0	1290 (22)	590 (456)	7.1

*N* number of cases, *CNS* Central Nervous System

### Spatial analysis

**Fig. 1:**
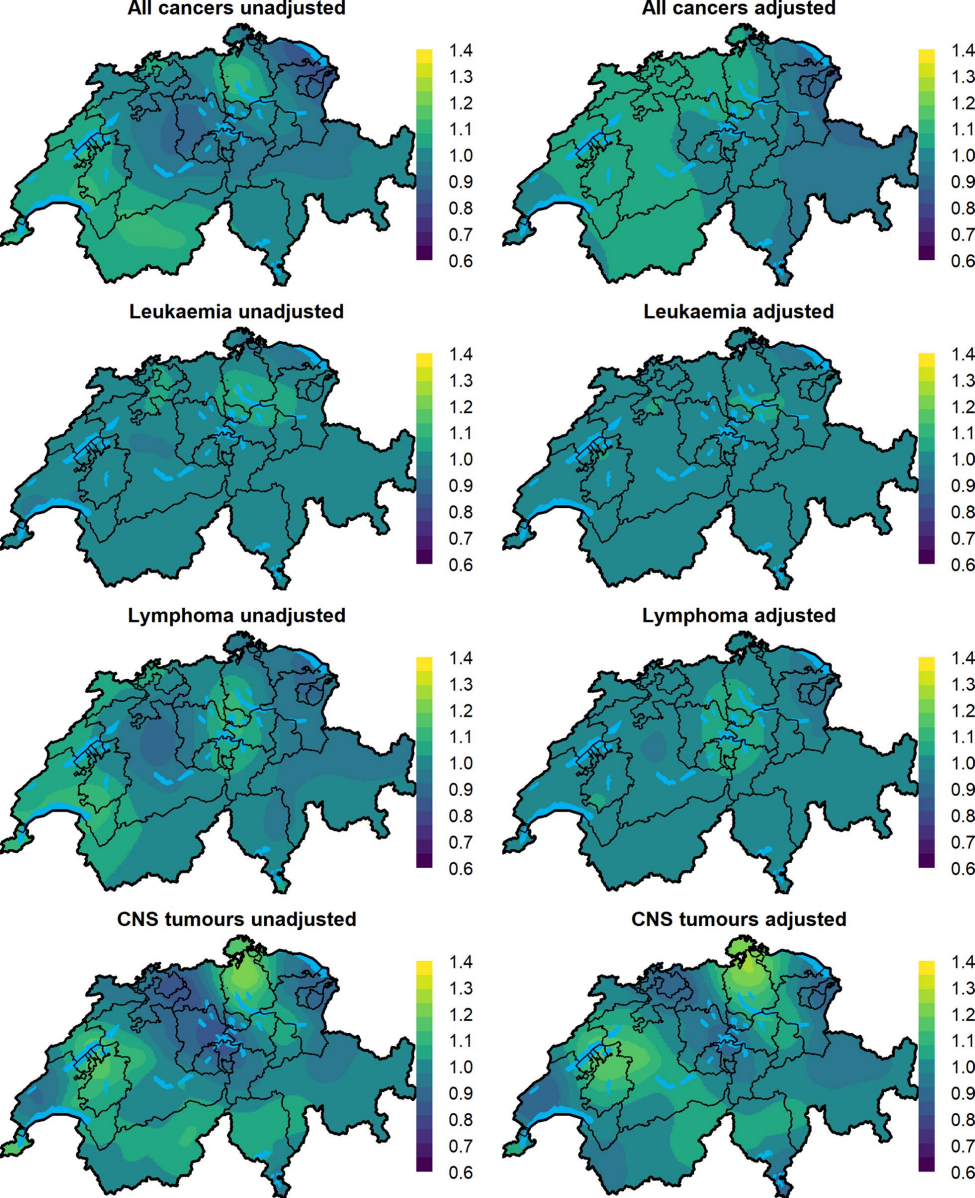
Maps of the median prosterior of the spatial relative risk for different cancer types during 1985–2015 in Switzerland. The adjusted models are models adjusted for predicted ambient NO_2_ concentration, predicted dose rate from terrestrial gamma and cosmic radiation, SEP, years of existing general cancer registry in the canton, language region and level of urbanisation

We found evidence of spatial variation for all cancers combined and CNS tumours at diagnosis, Fig. 1, Table 2 and Additional file 1: Table S2. For leukaemia and lymphoma, the posterior median of the variance hyperparameter of the Gaussian field ($\sigma^{2}$) was shrunk to 0 or values close to 0, indicating small, if any, spatial variation (Table 2 and Additional file 1: Table S2).

**Table 2:** Median posterior of the variance hyperparameter of the Gaussian field ($\sigma^{2}$) for the unadjusted and adjusted model, median posterior of variation explained ($V\left( {Z\left( s \right)} \right)$) and median posterior of grid specific relative risk based on residence at diagnosis

	LGCPs			
	All cancers	Leukaemia	Lymphoma	CNS tumours
$\sigma^{2}$ unadjusted^a^ (median, 95% CI)	0.01 (0, 0.02)	0.00 (0, 0.03)	0.01 (0, 0.04)	0.02 (0.01, 0.06)
$\sigma^{2}$ adjusted^b^ (median, 95% CI)	0.01 (0, 0.03)	0.00 (0, 0.01)	0.00 (0, 0.03)	0.02 (0, 0.06)
Variation explained^c^ (median; 95% CI)	0.72 (0.43, 0.89)	0.81 (0.58, 0.94)	0.82 (0.60, 0.94)	0.64 (0.31, 0.84)
RR unadjusted^a^ (median; range^d^)	0.99 (0.83, 1.13)	1.00 (0.96, 1.09)	0.99 (0.9, 1.13)	1.01 (0.82, 1.23)
RR adjusted^b^ (median; range^d^)	1.02 (0.86, 1.08)	1.00 (0.97, 1.04)	1.00 (0.96, 1.07)	1.00 (0.87, 1.25)

*CI* credibility intervals, *RR* grid specific relative risk compared to Switzerland as a whole, *LGCP* log-Gaussian Cox process, *CNS* Central and Nervous System

^a^The unadjusted model refers to the models without any covariates

^b^Adjusted for NO_2_, background radiation, years of general cancer registration, linguistic region and degree of urbanicity

^c^Variation explained by the covariates from the fully adjusted model, defined as $R^{2} = \frac{{V\left( {\varvec{X}\left( s \right)\varvec{\beta}} \right)}}{{V\left( {\varvec{X}\left( s \right)\varvec{\beta}} \right) + V\left( {Z\left( s \right)} \right)}}$ where $V\left( \cdot \right)$ denotes the variance over the $K$ spatial units, $\varvec{\beta}$ is the vector of intercept and covariates, $\varvec{X}$ the design matrix and $Z\left( s \right)$ the Gaussian field. The variation here refers to the fully adjusted model

^d^Range is defined as [min, max]

**Fig. 2:**
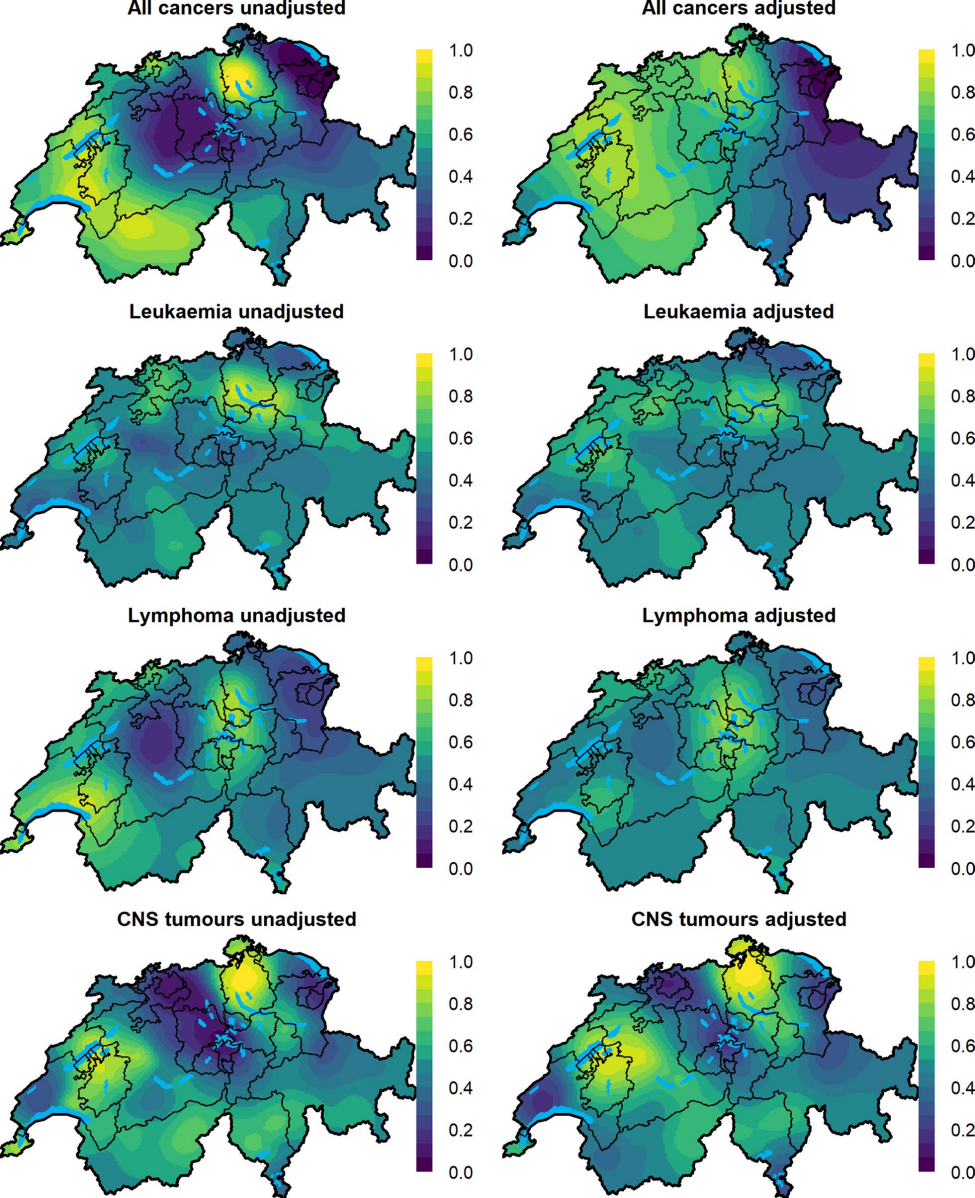
Maps of posterior probabilities that the spatial relative risk per grid cell is larger than 1 (exceedance probabilities) for different childhood cancers groups during 1985–2015 in Switzerland. The adjusted models are adjusted for predicted ambient NO_2_ air concentration, predicted dose rate from terrestrial gamma and cosmic radiation, SEP, duration in years of general cancer registration in the canton, language region and level of urbanisation

**Fig. 3:**
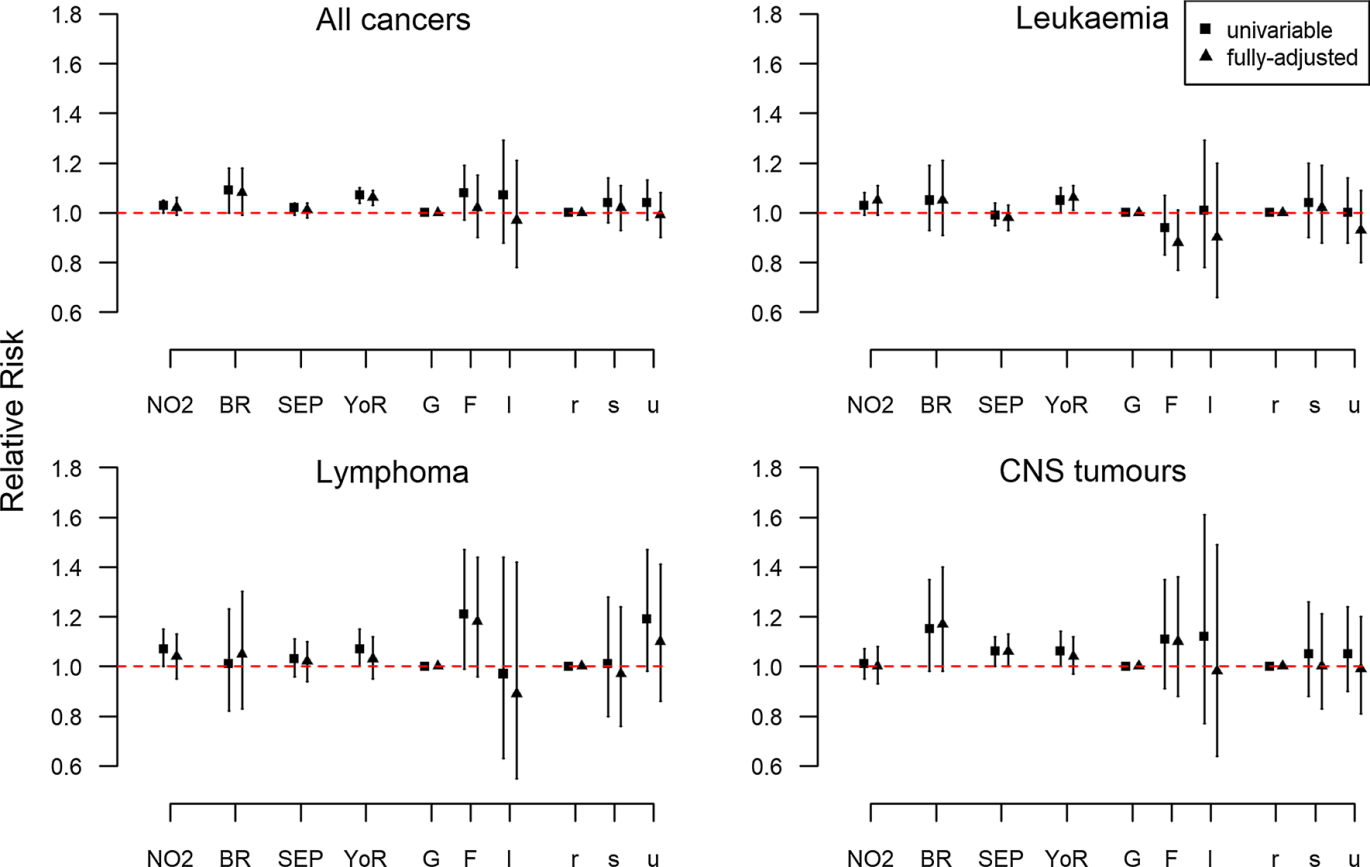
Univariable and fully adjusted regression analysis at time of diagnosis. The fixed effects are summarized using the posterior median of the relative risk together with 95% credibility regions. *NO*_*2*_ nitrogen dioxide, *CNS* Central Nervous System tumours, *BR* total dose background radiation, *SEP* socio-economic position, *YoR* years of existing cantonal registry, *G* German speaking part, *F* French speaking part, *I* Italian speaking part, *r* rural areas, *s* semi-urban areas, *u* urban areas. Predicted ambient NO_2_ air concentration, predicted background ionising radiation, SEP and duration in years of general cancer registration in the canton were scaled so that the standard deviations (SD) are 1 and considered as linear effects. Their interpretation is a multiplicative increase (or decrease) in the number of observed cases compared to the number of the expected cases per 1 SD increase (or decrease) in the covariate. The sd for predicted ambient NO_2_ air concentration is 77.7 μg/m^3^ × 10, for predicted background ionising radiation 60.2 ${\text{nSv}}/{\text{h}}$, for SEP 8.7 units and for duration in years of general cancer registration in the canton 11.6 years. The fully-adjusted models are models adjusted for predicted ambient NO_2_ air concentration, predicted dose rate from terrestrial gamma and cosmic radiation, SEP, duration in years of general cancer registration in the canton, language region and level of urbanisation

For all cancers grouped together, the medians of the posterior distributions of the unadjusted RR evaluated at the centroids of $1 \times 1\;{\text{km}}^{2}$ grid cells varied from 0.83 to 1.13 (min to max) throughout Switzerland, indicating at most a 13% increase in the risk in certain grid cells compared to Switzerland as a whole (Table 2 and Fig. 1). The corresponding exceedance probability maps show areas, for which the posterior probability of having an RR greater than 1 is above 0.80 highlighted in light green or yellow (Fig. 2). When we adjusted for the selected covariates almost 72% (95% CI 43%, 89%) of the observed variation was explained, with the median RR after adjustment varying from 0.86 to 1.08 (min to max), Fig. 1, Table 2. The putative risk factors explained 65% (35%, 86%) of the observed variation (Additional file 1: Table S3). In the fully adjusted model, the factors predicted ambient NO_2_ air concentration (RR 1.02; 95% CI 0.99–1.06 per 1 SD increase in NO_2_), predicted dose rate from terrestrial gamma and cosmic radiation (1.08; 0.99–1.18) duration in years of general cancer registration in the canton (1.06; 1.03–1.09) were positively associated with cancer risk, whereas the association with the other covariates was weak (Fig. 3 and Additional file 1: Table S4).

Childhood leukaemia risks showed smaller spatial variation with the median posterior RR per grid cell varying from 0.96 to 1.09 on the unadjusted and from 0.97 to 1.04 on the fully adjusted model, Fig. 1 and Table 2. The proportion of spatial variation explained by the selected covariates was 81% (58%, 94%), Table 2, whereas solely by the selected risk factor 64% (33%, 84%), Additional file 1: Table S3. In the fully adjusted model, the factors associated with the spatial risk of childhood leukaemia were predicted ambient NO_2_ air concentration exposure (1.05; 0.99–1.11) and duration in years of general cancer registration in the canton (1.06; 1.01–1.11), Fig. 3 and Additional file 1: Table S4.

A small amount of spatial variation of RR was also observed for childhood lymphoma with the median RR varying from 0.90 to 1.13 on the unadjusted model and 0.96 to 1.07 on the adjusted model (Fig. 1 and Table 2). About 82% (60%, 94%) of the observed spatial variation in the risk could be explained with the selected covariates, most of it due to the putative risk factors (Additional file 1: Table S3). In the fully adjusted model, the factor contributing most was living in the French speaking part of Switzerland with a 1.18 (0.96, 1.44) RR increase compared to living in the German speaking part, Fig. 3 and Additional file 1: Table S4.

Among the investigated diagnostic groups, the greatest spatial variation of cancer risks was observed for childhood CNS tumours. The median posterior grid-specific RR varied from 0.82 to 1.23 before adjusting, and from 0.87 to 1.25 after adjusting for the selected covariates. These covariates explained 64% (31%, 84%) of the observed spatial variation, and the putative risk factors alone 62% (28%, 92%), Additional file 1: Table S3 and Table 2. The adjusted RR was increased for the predicted exposure to background ionising radiation exposure (1.17; 0.98–1.40), SEP (1.06; 1.00–1.13) and duration in years of general cancer registration in the canton (1.04; 0.97–1.12). The association of the other covariates was weak, Fig. 3 and Additional file 1: Table S4.

We also examined the spatial variation of childhood cancers using place of birth. The spatial variation of cancer risks was generally smaller but the spatial patterns were largely consistent with the results for diagnosis (Additional file 1: Figures S10–S11 and Tables S3, S5).

We also examined the spatial variation using the BYM model. The maps and variation of median posterior RR were similar to the ones obtained by LGCPs, Additional file 1: Figures S10–S15. The estimates of the fixed effects were in the same direction but tended to be somewhat weaker than in the LGCP models (Additional file 1: Tables S4–S7).

### Sensitivity analysis

The resulting maps and effect estimates varied only little when using different priors for the hyperparameters, Additional file 1: Figures S16–S25.

### Post-hoc analysis

Given the larger spatial variation in the risk of CNS tumours we ran several post hoc analyses for this diagnostic group. First, we restricted the analysis to place of diagnosis for the period of 1995–2015 (n = 968), in which the coverage is highest (> 95%). The resulting spatial pattern was closely similar to the main analysis (Additional file 1: Figure S26). Second, we wanted to identify if the observed variation of CNS tumour was specific to particular diagnostic subgroups. We reran the analysis for place at diagnosis for astrocytoma (IIIb, n = 511 cases), intracranial and intraspinal embryonal tumours (IIIc, n = 266) and other CNS (IIIa, IIId–f, n = 512), following the classification used in our previous analysis of spatial clustering of childhood cancers in Switzerland [17]. We found that intracranial and intraspinal embryonal tumours showed the highest spatial variation with the median posteriors RR varying from 0.74 to 1.59 (min to max) in the unadjusted and 0.74 to 1.38 in the adjusted model, Additional file 1: Figure S27. However, the areas highlighted in Figs. 1, 2 stand out in all CNS subgroups Additional file 1: Figures S27, S28. Lastly, we hypothesized that differences in diagnostic practices between the nine SPOG clinics may explain the apparent spatial variation of CNS tumour risks. We thus constructed a spatial covariate reflecting the catchment areas of the different SPOG centres. Including an additional random effect to adjust for these catchment areas only slightly reduced the unexplained spatial variation. The spatial pattern of relative risk remained largely unchanged (Additional file 1: Text S3 includes the analysis and figures).

## Discussion

### Main findings

This nationwide study based on precise locations of residence sheds new light on the spatial variation of childhood cancer incidence in Switzerland and the extent to which this variation can be explained by environmental exposures and other spatial covariates. The spatial variation of cancer risk was small for childhood leukaemia and lymphoma and mostly explained by covariates. That of CNS tumours, particularly intracranial and intraspinal embryonal tumours, was larger and persisted after adjustment for covariates. Duration of general cancer registration in the canton was associated with higher observed cancer risk. Other covariates associated with cancer incidence included predicted ambient air concentration of NO_2_ for all cancers, lymphoma and leukaemia and SEP and modelled dose rates from terrestrial gamma and cosmic background radiation for CNS tumours and all cancers.

### Comparison of our study with other spatial analyses of childhood cancer risks

Compared to other studies that have investigated the spatial distribution of childhood cancers, our study stands out in that it uses precise geocoded place of residence and attempts to explain any spatial variation with commonly discussed putative environmental risk factors and completeness of registration. Our study is comparable with studies that performed parametric disease mapping, and in the lack of other studies that used LGCPs, with the previous studies that investigated the spatial variation of childhood leukaemia risks using areal data and BYM models in France [10], Yorkshire, UK [9] and in Florida, USA [12]. A study in France on acute leukaemia reported no evidence of spatial variation in the incidence of acute leukaemia at the département level [10]. A study in Yorkshire using data aggregated on the electoral ward level reported higher childhood leukaemia risks in the less populated county of North Yorkshire [9]. We did not observe higher leukaemia risk in less populated areas. Our results are in agreement with a study in Florida that reported evidence of spatial variation of brain tumours for cases 0–19 years old [12].

Other studies examining the spatial distribution of childhood cancer have focused on extra-Poisson variation and spatial clustering [34]. The general picture shows mixed results for childhood leukaemia and weak or no evidence of spatial clustering of lymphoma and CNS tumours [35–37]. In previous studies using the same data, we found no evidence of clustering of childhood cancers, leukaemia, lymphoma or CNS tumours, but weak evidence, consistent with the literature, for Hodgkin lymphoma and embryonal CNS tumours [38, 39]. We observed a cluster of intracranial and intraspinal CNS tumours in the French speaking part of Switzerland consistent with the pattern observed for CNS tumours in the present study [38].

### Comparison of our study with other studies on environmental risk factors of childhood cancer

The observed spatial associations between childhood cancer risks and putative risk factors are in broad agreement with other studies that have investigated these associations disregarding the spatial context.

Of the included covariates in the current study, predicted ambient NO_2_ air concentration showed the strongest spatial association with childhood leukaemia risks. There is increasing evidence of a link between traffic related air pollution and childhood cancers, in particular childhood leukaemia [40]. In recent meta-analyses associations with leukaemia risks were strongest for exposure to benzene and weaker for NO_2_ [3]. Using partly overlapping data, we reported an increased risk of leukaemia among children living less than 100 m from a highway [21].

Previous studies investigating childhood cancer risks in relation to background ionising radiation showed mixed results [22, 41–44]. While two studies reported associations between childhood leukaemia and gamma radiation [22, 41], others found no evidence of an association [42–44]. Using partly overlapping data, we previously reported evidence of associations with gamma radiation for both childhood leukaemia and CNS tumours [22]. In the current study the association was largest for all cancers and CNS tumours. The evidence from other studies examining the effect of gamma radiation on the risks of CNS tumours in children was weak [41, 43].

Our study found weak evidence of a potential association between SEP and CNS tumours. Previous studies in Switzerland have reported weak association between socioeconomic status and childhood leukaemia incidence, but a strong effect for CNS survival [45, 46]. Our results are consistent with a large UK case–control study which reported increased risk of CNS tumours in higher social classes [47]. A recent study in Spain also reported a positive association between risk of CNS tumours and socioeconomic status [48]. In contrast, a study in North-West England [49] and a study from Norway [50] found no evidence of an association between CNS tumours and measures of socio-economic status.

### Strengths and limitations

To the best of our knowledge this is the first study attempting to model and explain the spatial distribution of childhood cancers using precise locations of residence. We used LGCPs, which represent the current state of the art for modelling such point data of disease incidence and, as we have recently shown, outperform traditional methods in identifying high risk areas [17]. These models allowed us to incorporate spatial covariates and quantify their contribution to explaining the observed spatial variation. We also tried to disentangle variation attributed to registration completeness from variation due to putative risk factors. Furthermore, in contrast to previous studies, we examined both place of birth and diagnosis. Although results were closely similar, this comparison could potentially have revealed differences in time windows of susceptibility to different risk factors. The population at risk was retrieved from national censuses and cases from a nationwide registry with high completeness [19]. We attempted to correct for potential selection bias due to regional differences in case ascertainment by including duration of general cancer registration in the canton and, in post hoc analysis, SPOG centre catchment areas.

Due to data availability, we could not include all potential environmental risk factors discussed in the literature, for instance pesticide exposure. Furthermore, the spatial covariates included are subject to measurement errors and do not perfectly capture the spatial variation of residential exposures. We had little information about the magnitude of measurement errors, making it hard to propagate it in our modelling framework. Although we partly adjusted for differences in registration coverage, there may still be differences unaccounted for by our analyses. Our analysis is purely spatial and disregards and temporal or spatiotemporal variation. In contrast with previous work, which focused on spatiotemporal clustering [51], we focused on identifying spatial patterns that remain stable over comparably long time periods. Such patterns potentially reflect stationary sources of relevant environmental exposures. We implicitly adjusted for the national time trends of childhood cancers using a time varying offset. But our analysis ignores any temporal or spatiotemporal trends of the selected covariates (e.g. NO_2_). This may have diluted their effects and biased the estimated coefficients towards the null. Such bias may have particularly affected estimates for traffic related air pollution, as NO_2_ concentration decreased considerably during the study period while other covariates including, SEP, urbanisation and background radiation were relatively stable. Potential avenues of future research include extending this analysis to incorporate full spatiotemporal risk factors and interactions.

### Interpretation of findings

Although the overall completeness of SCCR is larger than 95% after the mid-90s [19], we found that duration of general cancer registration in the canton can influence the apparent spatial variation of childhood cancers based on data from SCCR. This suggests that there are regional differences in registration completeness, which should be accounted for in future aetiological studies in Switzerland.

Our results are suggestive of an environmental aetiology for childhood CNS tumours and of aetiological differences between their histological subtypes. In post hoc analyses, the observed spatial variation was not fully explained by differences in cancer registration in the early years of the SCCR as it persisted in the more recent periods. Neither did differences between SPOG centres, for instance in ascertainment practices, explain the spatial variation. Unmeasured environmental risk factors are thus a likely explanation of the observed spatial variation. Possibly, spatial differences in the prevalence of genetic syndromes associated with these tumours might also partially explain the observed variation. In future research, there should be increased attention on putative environmental risk factors of CNS tumours, including SEP, background radiation and pesticide exposure (which was not accounted for in our analyses).

Our analysis shows that, locally, risks for childhood cancer in Switzerland can deviate from the national level by up to 13% (range of RR: 0.83–1.13). These deviations tend to be smaller (0.96–1.09) for childhood leukaemia, in which case they appear to be largely explained by spatial covariates included in analysis. In contrast, for CNS tumours these variations are larger (0.82–1.23) and to a lesser extent explained by included covariates. Adjustments for potential regional differences in registration or diagnostic practices did materially reduce this variability. If indeed this variation was caused by environmental factors, similar regional variation is to be expected in other countries. While our analysis could not identify the source of this variation, it does suggest that environmental factors other than background radiation and traffic related pollution is driving geographic variation in childhood CNS tumour incidence. Future research should therefore focus on investigating the environmental aetiology of CNS tumours. Such research could benefit from separate analysis of histological subtypes and the pooling of data across different studies to increase statistical power.

## Conclusion

This study provides evidence of spatial differences in the incidence of childhood CNS tumours in Switzerland that could be partially explained by variations in socio-economic factors and natural background radiation. The spatial variation of the risks for childhood leukaemia and lymphoma was smaller and mostly explained by measured covariates. Our study provides further support for an environmental aetiology for childhood CNS tumours, highlighting the need for future studies to distinguish between histologic subtypes.

## Supplementary information

**Additional file 1.** Supplementary text, tables and figures supporting the main manuscript.

## Data Availability

The Swiss Childhood Cancer Registry is the permanent repository of data on childhood cancer cases used in this study. This data cannot be made publicly available for both legal and ethical reasons as this would compromise patient confidentiality and participant privacy. Interested researchers may contact the corresponding author or the Swiss Childhood Cancer Registry (http://childhoodcancerregistry.ch/) via its online contact form for further information.

## Abbreviations

CNS: Central nervous system
SCCR: Swiss childhood cancer registry
SNC: Swiss national cohort
ICCC3: International classification of childhood cancers 3rd edition
NO_2_: Nitrogen dioxide
SEP: Socioeconomic position
SPOG: Swiss paediatric oncology group
LGCP: Log-Gaussian Cox process
RR: Relative risk
SD: Standard deviation
BYM: Besag-York-Mollié
