# Supplementary material for: Bayesian spatial modelling of childhood cancer incidence in Switzerland using exact point data: a nationwide study during 1985–2015
Source: Int J Health Geogr. 2020 Apr 17;19:15. doi: 10.1186/s12942-020-00211-7 (PMC7165384; doi:10.1186/s12942-020-00211-7)
Supplement: Supplementary file 1 — Additional file 1. Supplementary text, tables and figures supporting the main manuscript. [file 12942_2020_211_MOESM1_ESM.pdf]

Additional File

Bayesian spatial modelling of childhood cancer incidence in Switzerland using exact point data: A nationwide study during 1985-2015

Authors: Garyfallos Konstantinoudis[1,2], Dominic Schuhmacher[3], Roland A Ammann[4], Tamara Diesch[5], Claudia E Kuehni[1], Ben D Spycher[1] for the Swiss Paediatric Oncology Group and the Swiss National Cohort Study Group

Affiliations:

1 Institute of Social and Preventive Medicine (ISPM), University of Bern, Bern, Switzerland.

2 Epidemiology and Biostatistics department, School of Public Health, Imperial College London, United Kingdom

3 Institute for Mathematical Stochastics, University of Göttingen, Germany

4 Department of Paediatrics Inselspital, Bern University Hospital, University of Bern, Bern, Switzerland.

5 University Children's Hospital Basel, Division of Paediatric Oncology/Haematology, Basel, Switzerland.

Corresponding author:

Garyfallos Konstantinoudis,

Epidemiology and Biostatistics department, School of Public Health, Imperial College London, United Kingdom

E-mail: g.konstantinoudis@imperial.ac.uk

Table of contents

Text S1. Calculation of population at risk and expected number of cases 4

Text S2. Model Formulation for the Besag-York-Mollié model 6

Text S3. SPOG areas 8

Table S1. Description of the selected covariates.. 12

Table S2. Spatial variation of childhood cancer risks based on residence at birth.. 13

Table S3. Variation explained by the selected covariates.. 14

Table S4. Median and 95% credibility regions of the posterior of risk ratios from spatial regression analysis using a log-Gaussian Cox process model for residence at diagnosis...................................... 15

Table S5. Median and 95% credibility regions of the posterior of risk ratios from spatial regression analysis using log-Gaussian Cox process model for residence at birth. 17

Table S6. Median and 95% credibility regions of the posterior of risk ratios from spatial regression using a Besag-York-Mollié model for residence at diagnosis. 19

Table S7. Median and 95% credibility regions of the posterior of risk ratios from spatial regression using Besag-York-Mollié model for residence at birth. 21

Figure S1. Expected number of cancers cases Ek adjusted by age group (0-4, 5-9, 10-15) and year of diagnosis per municipality, see Text S1 for definition. 23

Figure S2. Expected number of cancers cases Ek adjusted by age group (0-4, 5-9, 10-15) and year of diagnosis per $1km\times 1km$ grid cell, see Text S1 for definition... 23

Figure S3. Modelled ambient NO_2 $[\mu g/m3\times 1000]$ air concentration per municipality and on a 500x500 grid.. 24

Figure S4. Modelled exposure to background ionising radiation $[nSv/h]$ from cosmic and terrestrial radiation per municipality and on a $2\times 2km2$ grid.. 25

Figure S5. Swiss socioeconomic (SEP) index as mean per municipality and a combination of municipality mean and 500x500$m2$ grid means.. 26

Figure S6. Duration in years of general cancer registration in the canton for period 1985-2015......... 27

Figure S7. Language regions per municipality in Switzerland. 28

Figure S8. Levels of urbanisation in Switzerland. 29

Figure S9. Autoregressive processes of order 1 to examine a more flexible fit for background ionising radiation, ambient NO_2, and socio-economic position. 30

Figure S11. Exceedance probability surfaces based on log-Gaussian Cox processes (LGCPs) and residence at birth. 31

Figure S12. Modelled relative risk surfaces based on Besag-York-Mollié (BYM) and residence at diagnosis.. 33

Figure S13. Exceedance probability surfaces based on Besag-York-Mollié (BYM) and residence at diagnosis. 34

Figure S14. Modelled relative risk surfaces based on Besag-York-Mollié (BYM) and residence at birth. 35

Figure S15. Exceedance probability surfaces based on Besag-York-Mollié (BYM) and residence at birth. 36

Figure S16. Sensitivity of relative risk surfaces of all cancers using log-Gaussian Cox processes, the unadjusted model and different priors for the range parameter. 37

Figure S17. Boxplots of relative risk surfaces of all cancers using log-Gaussian Cox processes, the unadjusted model and different priors for the range parameter. 38

Figure S18. Sensitivity of relative risk surfaces of all cancers using log-Gaussian Cox processes, the fully adjusted model and different priors for the range parameter. 39

Figure S19. Boxplots of relative risk surfaces of all cancers using log-Gaussian Cox processes, the fully adjusted model and different priors for the range parameter. 40

Figure S20. Regression coefficients of all cancers using log-Gaussian Cox processes, the fully adjusted model and different priors for the range parameter. 41

Figure S21. Sensitivity of relative risk surfaces of CNS tumours using log-Gaussian Cox processes, the unadjusted model and different priors for the range parameter. 42

Figure S22. Boxplots of relative risk surfaces of CNS tumours using log-Gaussian Cox processes, the unadjusted model and different priors for the range parameter.. 42

Figure S23. Sensitivity of relative risk surfaces of CNS tumours using log-Gaussian Cox processes, the fully adjusted model and different priors for the range parameter 44

Figure S24. Boxplots of relative risk surfaces of CNS tumours using log-Gaussian Cox processes, the fully adjusted model and different priors for the range parameter 45

Figure S25. Regression coefficients of CNS tumours using log-Gaussian Cox processes, the fully adjusted model and different priors for the range parameter. 46

Figure S26. Post-hoc analysis for CNS tumours restricting to cases (n=968) diagnosed during 1995-2015. 47

Figure S27. Relative risk surfaces of subgroups of CNS tumours 48

Figure S28. Exceedance probability surfaces of subgroups of CNS tumours 48

References 50

Text S1. Calculation of population at risk and expected number of cases

Population data was available through the Swiss National Cohort (SNC) including age, sex and geocoded location of residence for all Swiss residents at time of census (i.e. 1990, 2000 and 2010-15). We could thus calculate exact age specific population counts for the years 1990, 2000, 2010, 2011, 2012, 2013, 2014 and 2015. We also obtained projected annual total population (all ages combined) by municipality from the Federal Statistical Office (FSO, https://www.bfs.admin.ch). To construct an age and year specific population at risk we performed the following procedure:

Let $P_{i,j,m}$ be the population in the i-th age group, the j-th year and the m-th municipality, and let $P_{*,j,m} = \sum_i P_{i,j,m}$, which is known for all years from FSO.

1. Calculate weights $w_{i,j,m} = \frac{P_{i,j,m}}{P_{*,j,m}}$ for $j = 1990, 2000$ & 2010 using data from the censuses.
2. To calculate the weights for the rest of the years perform a linear interpolation:

$$w_{i,j,m} = \begin{cases} w_{i,1990,m} \;\; for\, j = 1985, \dots, 1989 \\ \frac{2000-j}{10} \cdot w_{i,1990,m} + \frac{j-1990}{10} \cdot w_{i,2000,m} \;\; for\, j = 1991, \dots, 1999 \\ \frac{2010-j}{10} \cdot w_{i,2000,m} + \frac{j-2000}{10} \cdot w_{i,2010,m} \;\; for\, j = 2001, \dots, 2009. \end{cases}$$

3. Calculate the interpolated population by municipality and year as: $P_{i,j,m} = w_{i,j,m} \cdot P_{*,j,m}$.

We followed a similar procedure to calculate the population denominator on a fine grid:

Let $P_{i,j,g}$ be the population in the i-th age group, the j-th year and the g-th grid cell, and let $P^{(m)}_{*,j,*} = \sum_{g \sim m} \sum_i P_{i,j,g}$, where $g \sim m$ denotes indices g of grid cells whose centroids lie in the m-th municipality.

1. Calculate weights $w_{i,j,g} = \frac{P_{i,j,g}}{P^{(m)}_{*,j,*}}$ for $j = 1990, 2000$ & 2010 using data from the censuses.
2. To calculate the weights for the rest of the years perform a similar linear interpolation as above:

$$w_{i,j,g} = \begin{cases} w_{i,1990,g} \;\; for\, j = 1985, \dots, 1989 \\ \frac{2000-j}{10} \cdot w_{i,1990,g} + \frac{j-1990}{10} \cdot w_{i,2000,g} \;\; for\, j = 1991, \dots, 1999 \\ \frac{2010-j}{10} \cdot w_{i,2000,g} + \frac{j-2000}{10} \cdot w_{i,2010,g} \;\; for\, j = 2001, \dots, 2009. \end{cases}$$

3. Calculate the interpolated population per grid as: $P_{i,j,g} = w_{i,j,g} \cdot P^{(m)}_{*,j,*} \approx w_{i,j,g} \cdot P_{*,j,m}$.

In this way, we obtain population estimates by age group, year and spatial unit (municipality or grid). To adjust for age and year in the models, we used an indirect standardization method. We did not adjust for sex because we have no reason to believe that there are discrepancies in the spatial distribution of girls and boys.

The expected number of cases in the k-th spatial unit (municipality or grid cell) and d-th diagnostic group (all cancers, leukaemia, lymphoma and CNS tumours), adjusted for year and age category, was calculated as:

$$E_{k,d} = \sum_i \sum_j q_{i,j,d} \cdot P_{i,j,k},$$

where $q_{i,j,d} = \frac{Y_{i,j,d}}{P_{i,j,*}}$. In the latter expression $Y_{i,j,d}$ is the disease count of the d-th diagnostic group, the i-th age category and the j-th year, and $P_{i,j,*} = \sum_k P_{i,j,k}$. We repeated this procedure for the different spatial units k and diagnostic groups d.

To calculate the expected number of cases at place of birth we repeated the above procedure but restricted to children <1 years old at census. The expected number of cases in the k-th spatial unit (municipality or grid cell) and d-th diagnostic group can be written as:

$$E_{k,d} = \sum_j q_{<1,j,d} \cdot P_{<1,j,k},$$

where $q_{<1,j,d} = \frac{Y_{*,j,d}}{P_{<1,j,*}}$. In the latter expression $Y_{*,j,d}$ is the disease count of the d-th diagnostic group for which we have a geocode of residence at birth available, and j is the year of birth. The denominator $P_{<1,j,*}$ is the total population of children <1 years old, in the j-th year in Switzerland. We calculated the expected number of cases per spatial unit adjusted for year and age and plugged them into the LGCP and BYM models as populations at risk e(s) and E_i, respectively, see main paper and Text S2.

Text S2. Model Formulation for the Besag-York-Mollié model

Let W be an observation window divided in spatial units $A_1, A_2, \dots, A_n$ (in the main analysis these units are the municipalities). Let Y_i for $i = 1, \dots, n$ be the disease counts, E_i the population at risk counts and r_i the relative risk in the i-th spatial unit. The model assumptions are $Y_i \sim Poisson(r_i E_i)$ for $i = 1, \dots, n$ and, writing $\boldsymbol{r} = (r_1, \dots, r_n)^T$,

$$\log(\boldsymbol{r}) = \boldsymbol{X\beta} + \boldsymbol{v} + \boldsymbol{u}$$
$$\boldsymbol{u} \sim N(0, \tau_1^{-1}\boldsymbol{Q}^{-1})$$
$$\boldsymbol{v} \sim N(0, \tau_2^{-1}\boldsymbol{I})$$
$$\boldsymbol{\beta} \sim N(0, b\boldsymbol{I}),$$

where $\boldsymbol{\beta} = (\beta_0, \beta_1, \dots, \beta_l)^T$ is a vector of covariates, **X** the model matrix, τ_1, τ_2 random precisions, b a large constant, and $\boldsymbol{I}$ the $n\times n$ identity matrix. The symbol $\boldsymbol{Q}^{-1}$ denotes the generalized inverse of the precision matrix $\boldsymbol{Q}$, which is specified to have entries

$$Q_{ij} = \begin{cases} n_{\delta_i}, & i = j \\ -1, & i \sim j \\ 0, & otherwise, \end{cases}$$

where $i \sim j$ indicates that spatial units A_i and A_j are first order neighbours and n_{δ_i} is the number of first order neighbours of unit A_i.

The above formulation denotes the BYM model as suggested in [1]. However, there are certain methodological limitations in this formulation. The precision matrix depends on the graph structure and thus the marginal variances are not comparable across different graphs. This problem can be bypassed by scaling the precision matrix [2, 3]. In addition, the precision parameters τ_1 and τ_2, even though they cannot be considered independent, are treated as independent in the prior specification. All these issues are bypassed by reparametrizing the model as

$$\log(\boldsymbol{r}) = \boldsymbol{X\beta} + \frac{1}{\sqrt{\tau}}\left(\sqrt{1-\phi}\boldsymbol{v} + \sqrt{\phi}\boldsymbol{u}^*\right)$$
$$\boldsymbol{u}^* \sim N(0, \boldsymbol{Q}_*^{-1})$$
$$\boldsymbol{v} \sim N(0, \boldsymbol{I})$$
$$\boldsymbol{\beta} \sim N(0, b\boldsymbol{I}),$$

where now $\boldsymbol{Q}_*$ is a scaled precision matrix so that the marginal variances are approximately equal to $(1-\phi)/\tau + \phi/\tau$, see [4] for details on the procedure. In the above formulation there is only one (random) precision parameter τ and an additional random mixing parameter $\phi \in [0,1]$, which describes the proportion of the marginal variance explained by the structured effect, i.e. a value of 0 indicates that the variation of the latent field is solely due to the unstructured (or overdispersion) component $\boldsymbol{v}$ and a value of 1 that the variation of the latent field is solely due to the structured component $\boldsymbol{u}^*$.

To complete the Bayesian model formulation, we use priors that penalise an increase of complexity compared to a base model (PC priors). More precisely, a constant decay rate (exponential decay) in the

Kullback-Leibler divergence between the more flexible and the base model is used, see [5]. In our case the base model for τ assumes no random effect, i.e. $\tau = \infty$, and the base model for ϕ assumes for given precision that there is no spatial dependence, i.e. $\phi = 0$. According to [5] the PC prior for τ is a type-2 Gumbel distribution:

$$\pi(\tau) = \frac{\theta}{2}\tau^{-3/2}\exp\left(-\theta\tau^{-1/2}\right),$$

where θ is a constant defined by investigators. In our case the selection of θ was based on the intuition that the log relative risk in a fixed area should be unlikely to have variance larger than 1. The PC prior of ϕ depends on the underlying graph structure and was computed in [5]. The user-defined scale of this prior was chosen so that the probability of being less than 0.5 is 0.5, reflecting that we have little knowledge on the mixing parameter.

The full model specification in the Bayesian framework reads then:

$$\log(\boldsymbol{r}) = \boldsymbol{X\beta} + \frac{1}{\sqrt{\tau}}\left(\sqrt{1-\phi}\boldsymbol{v} + \sqrt{\phi}\boldsymbol{u}^*\right)$$

$$\boldsymbol{u}^* \sim N(0, \boldsymbol{Q}_*^{-1})$$

$$\boldsymbol{v} \sim N(0, \boldsymbol{I})$$

$$\boldsymbol{\beta} \sim N(0, b\boldsymbol{I})$$

$$\tau \sim PCprior(0.01, 1)$$

$$\phi \sim PCprior(0.5, 0.5).$$

Text S3. SPOG areas

In a post-hoc analysis, we adjusted models for CNS tumours for potential geographical variation due to differences in practices between the nine SPOG clinics in which patients were diagnosed (see Figure S3.1 for the location of the SPOG clinics). An example of such discrepancies might be differences in the way SPOG clinics perform MRIs or CT scans. If we hypothesize that a specific SPOG clinic is more likely to perform an MRI for potential cases, then it is more likely to identify CNS tumours that in other cases would be identifiable only later in life. In order to adjust for such discrepancies, we created the SPOG areas.

We calculated the proportion of cases in the *i-th* medstat region (a spatial unit constructed based on postal code boundaries and uniform population size, for more information see: https://www.bfs.admin.ch/bfs/de/home/statistiken/gesundheit/nomenklaturen/medsreg.html) who were diagnosed diagnosed/reported in the *j-th* SPOG clinic.

Figure S3.2 shows these probabilities by medstat region in Switzerland. We observe that the SPOG clinics in Bern, Lausanne and Zurich cover most of Switzerland. In addition, we observe some overlap of catchment areas, in particular for Bellinzona, Aarau and Lucerne with other SPOG centres. We defined catchment areas for each SPOG centre based on the highest probabilities of allocation while enforcing these areas to be contiguous (no islands permitted; Fig3.3). To validate this decision, we created the Voronoi tessellation of the cases over the domain. The centroid of each Voronoi polygon is thus the geocode of a cancer case. The boundaries of the i-th polygon are defined as follows: for every point inside the i-th polygon the distance between this point and the centroid of the i-th polygon is smaller than the distance of this point and any other polygon's centroid. For every case, we thus have a polygon and we can flag its area based on the SPOG clinic at which the case was diagnosed. This map serves as a "ground truth" against which the constructed catchment areas can be compared.

Figure S3.1. Specialized childhood oncology clinics or SPOG clinics in Switzerland (red boxes). The SPOG clinics are located in: Aarau, Bellinzona, Basel, Geneva, Zurich, St Gallen, Lausanne, Bern, Lucerne.

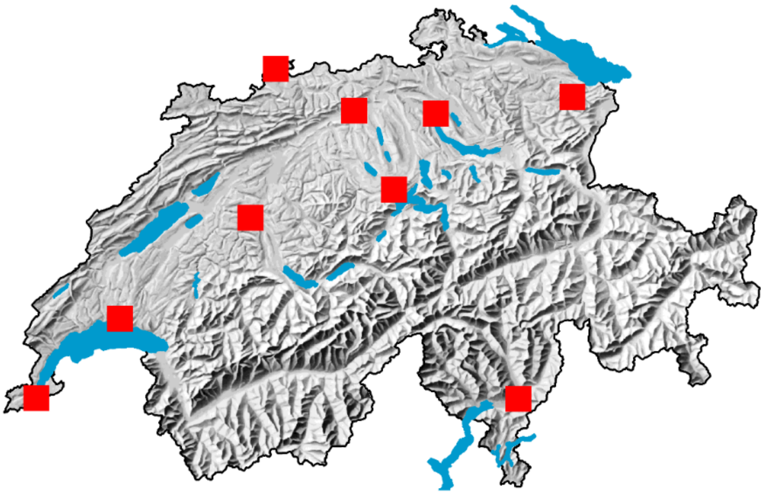

Figure S3.2. Maps of probabilities that cases residing in medstat (regional units based on post-code boundaries and uniform population across Switzerland) region i will be reported in SPOG clinic j.

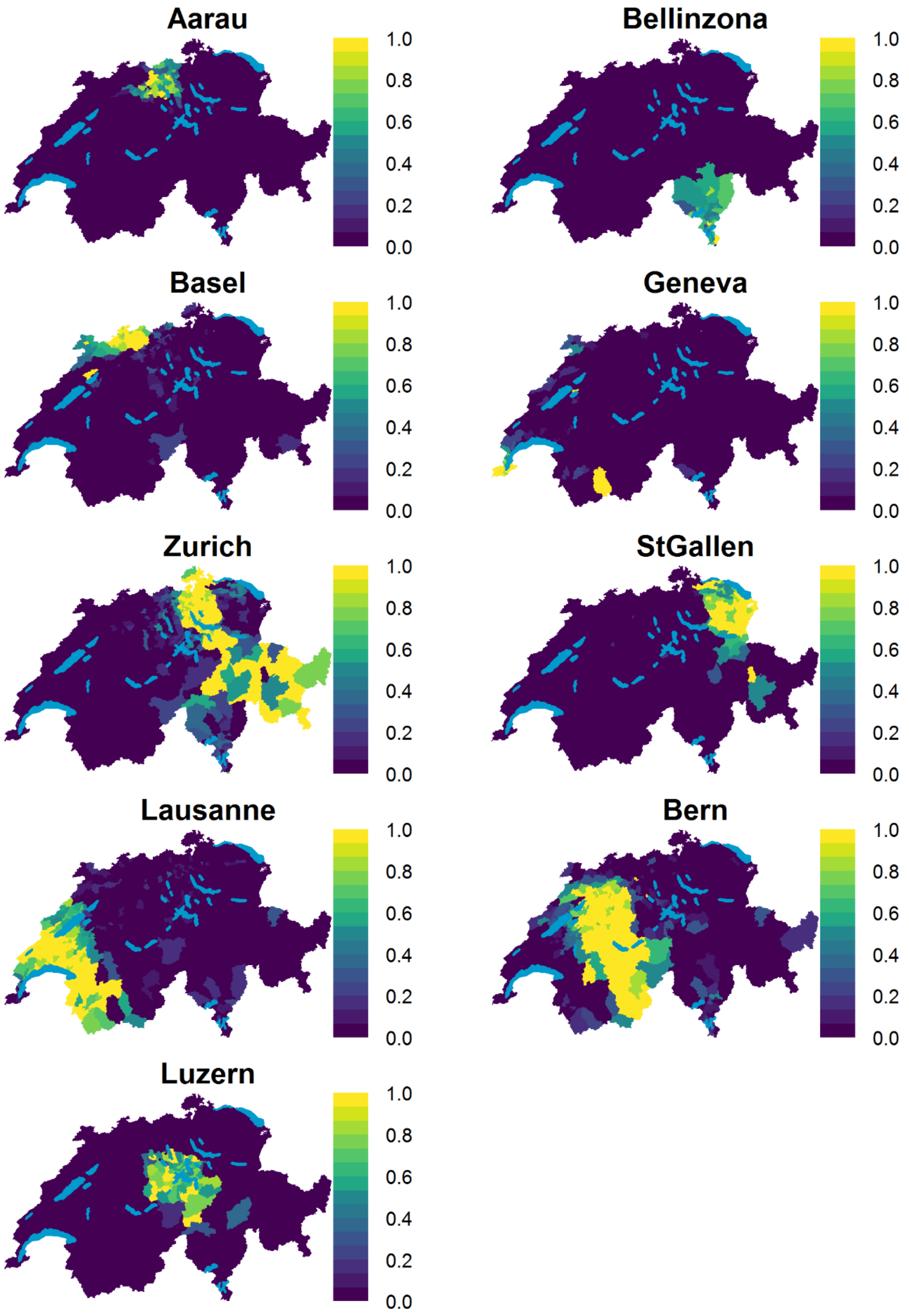

Figure S3.3. SPOG regions based on the medstats clinic with the highest probability as defined in Figure S37. We used information from neighbouring medstats to deal with noise.

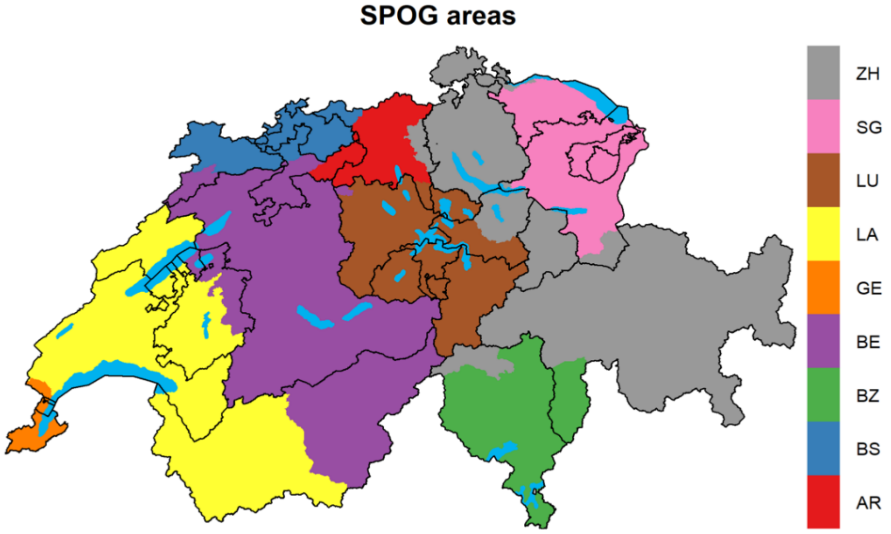

We then reran the fully adjusted model for CNS tumours at place of diagnosis, by adding a random intercept for SPOG catchment areas as defined above. The model specification using the notation of Text S2 is:

$$\log r(s) = \boldsymbol{X}(s)\boldsymbol{\beta} + Z_*(s) + \boldsymbol{\omega}_l \mathbf{1}\{s \in S_l\}$$

$$\boldsymbol{Z} \sim N(\mathbf{0}, \boldsymbol{Q}(\rho, \sigma)^{-1})$$

$$\boldsymbol{\omega} \sim N(0, \sigma_1^2 \boldsymbol{I})$$

$$\boldsymbol{\beta} \sim N(0, b\boldsymbol{I})$$

$$\sigma, \sigma_1 \sim PCprior(0.01, 1)$$

$$\rho \sim PCprior(0.5, 60)$$

where $\boldsymbol{\omega}_l = (\omega_1, \dots, \omega_9)^T$ are the random intercepts for the SPOG catchment areas, σ_1 is their standard deviation and $\mathbf{1}\{s \in S_l\}$ is equal to 1 if location s is within the l-th SPOG region and 0 otherwise. As in our main LGCP, the variance b of the fixed effects was set to 10. We observed some variation of risk at SPOG region level, with the median posterior and 95% CI of the variance of the random intercept being 0.01 (0.001, 0.10). The SPOG specific median posterior RR ($\exp(w_l)$) ranged from 0.88-1.14 (min to max). The value of 1.14 was observed in the Zurich SPOG area, probably driven by the increased risk in the canton of Zurich and Schaffhausen (top panel Figure S3.4). The grid specific median posteriors RR ranged from 0.91 to 1.12 (min to max) and were thus somewhat less variable compared to the model without adjustment for SPOG catchment areas (Table S1). However, the highlighted areas of increased risk remained the same, see middle and bottom panel of Figure S3.4.

Figure S3.4. Post-hoc analysis showing the median SPOG specific relative risk (RR) for top panel, grid-specific relative risk (RR), middle panel, and exceedance probabilities, i.e. Pr(RR >1); bottom panel, of childhood CNS tumours using the place of diagnosis, the fully adjusted model, plus a random intercept on the Swiss paediatric oncology group region level, see bottom panel of Figure S3.3.

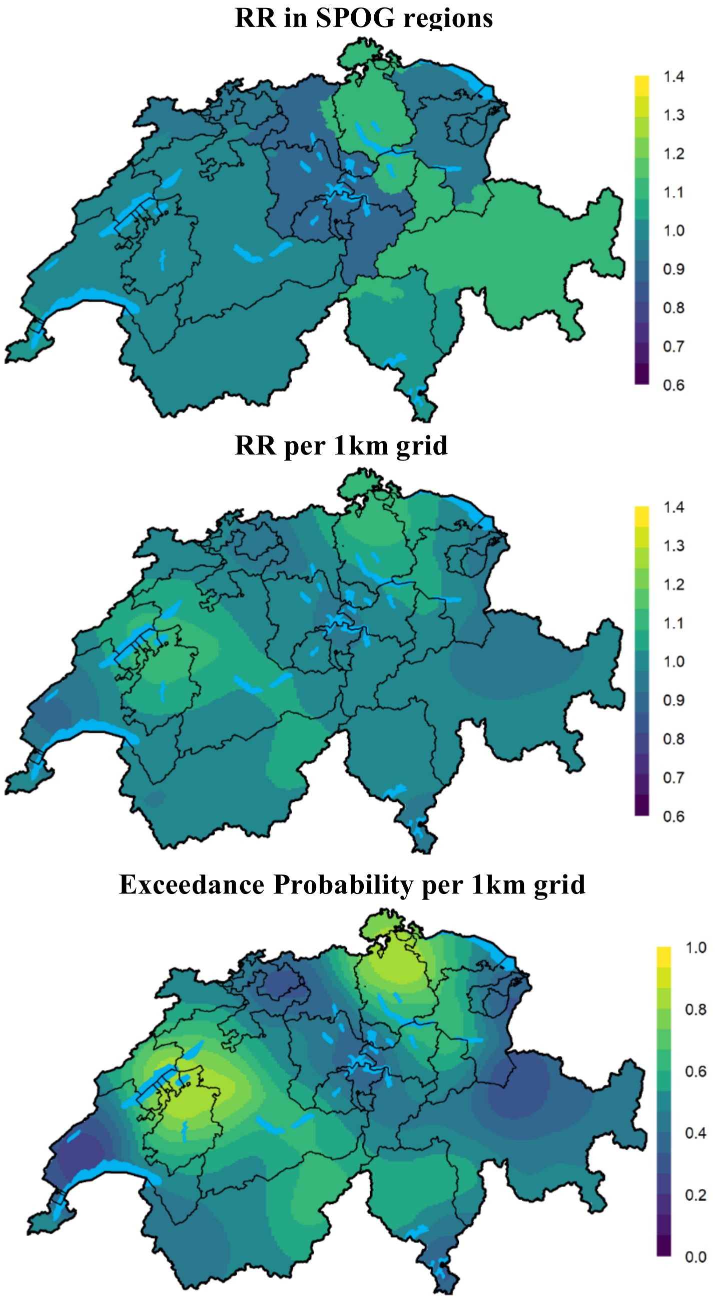

Table S1. Description of the selected covariates.

Variable	Unit	Standard Deviation (SD)		Type	Spatial unit	Year	source
		1x1 km^2	municipal				
NO_2 (Figure S3)	$\mu g/m^3$ $\times 10$	77.7	60.9	continuous	200x200 m^2	1990, 2000, 2010	Meteotest
total radiation (Figure S4)	nSv/h	60.2	37.5	continuous	2x2km^2	1960 - 1995	[6, 7]
Swiss-SEP (Figure S5)	index	8.7	8.3	continuous	building level	2000	[8]
cantonal registry (Figure S6)	Years [y]	11.6	12.4	continuous	canton	2015	http://www.nicer.org/
language region (Figure S7)	-	-	-	German (baseline), French, Italian	municipal	2012	Federal Office of Statistics
Urbanisation level (Figure S8)	-	-	-	rural (baseline), semi-urban, urban	municipal	2012	Federal Office of Statistics

Abbreviations NO_2: Nitrogen Dioxide, SEP: Socio-economic position

Table S2. Spatial variation of childhood cancer risks based on residence at birth. Median posterior variance, variation explained and grid specific relative risk from the unadjusted and the adjusted models.

	All cancers	Leukaemia	Lymphoma	CNS tumours
σ^2unadjusted[a]	0.00	0.00	0.01	0.01
(median, 95% CI)	(0, 0.01)	(0, 0.03)	(0, 0.05)	(0, 0.05)
σ^2adjusted[b]	0.00	0.00	0.01	0.01
(median, 95% CI)	(0, 0.02)	(0, 0.02)	(0, 0.08)	(0, 0.05)
Variation	0.86	0.77	0.80	0.67
explained[c]	(0.61, 0.97)	(0.54, 0.93)	(0.51, 0.93)	(0.38, 0.86)
(median; 95% CI)				
RR unadjusted[a]	1.00	1.00	1.00	1.00
(median; Range[d])	(0.89, 1.06)	(0.96, 1.05)	(0.92, 1.1)	(0.90, 1.11)
RR adjusted[b]	1.01	1.00	1.00	1.00
(median; Range[d])	(0.9, 1.06)	(0.95, 1.06)	(0.94, 1.14)	(0.90, 1.19)

Abbreviations: CI: credibility intervals, RR: grid specific relative risk compared to Switzerland as a whole, LGCP: log-Gaussian Cox process, CNS: Central and Nervous System

[a] the unadjusted model refers to the models without any covariates but indirectly standardised for year of birth

[b] adjusted for modelled ambient NO_2 air concentration (Figure S3), modelled exposure to background ionising radiation (Figure S4), Swiss socioeconomic index (Figure S5) duration in years of general cancer registration in the canton (Figure S6), linguistic region (Figure S7) and degree of urbanicity (Figure S8)

[c] variation explained by the covariates; $R^2 = \frac{V(\boldsymbol{X}(s)\boldsymbol{\beta})}{V(\boldsymbol{X}(s)\boldsymbol{\beta})+\mathrm{V}(\mathrm{Z(s)})}$ using $V(\cdot)$ to denote the variance over the K spatial units, $\boldsymbol{\beta}$ is the vector of intercept and covariates, $\boldsymbol{X}$ the design matrix and Z(s) the Gaussian field

[d] Range is defined as [min, max]

Table S3. Variation explained by the selected covariates. Posterior median and 95% credibility regions for the variation explained by all the covariates, the potential risk factors (NO_2, background radiation, linguistic region and degree of urbanicity), and the factors influencing the completeness of the registry (years of cantonal cancer registration).

	All cancers	Leukaemia	Lymphoma	CNS tumours
**Birth**				
Variation explained[a]: full model	0.86 (0.61, 0.97)	0.77 (0.54, 0.93)	0.80 (0.51, 0.93)	0.67 (0.38, 0.86)
Variation explained[a]: full model without YoR	0.89 (0.71, 0.95)	0.65 (0.31, 0.87)	0.79 (0.50, 0.92)	0.68 (0.39, 0.88)
Variation explained[a]: univariable with YoR	0.12 (0, 0.48)	0.55 (0, 0.81)	0.08 (0, 0.44)	0.06 (0, 0.40)
**Diagnosis**				
Variation explained[a]: full model	0.72 (0.43, 0.89)	0.81 (0.58, 0.94)	0.82 (0.60, 0.94)	0.64 (0.31, 0.84)
Variation explained[a]: full model without YoR	0.65 (0.35, 0.86)	0.64 (0.33, 0.84)	0.83 (0.63, 0.93)	0.62 (0.28, 0.92)
Variation explained[a]: univariable with YoR	0.50 (0.25, 0.72)	0.35 (0.01, 0.70)	0.34 (0, 0.64)	0.15 (0, 0.45)

Abbreviations: CNS: Central and Nervous System, YoR: Year of cantonal cancer registration (Figure S6)

[a] variation explained by the covariates; $R^2 = \frac{V(\boldsymbol{X}(s)\boldsymbol{\beta})}{V(\boldsymbol{X}(s)\boldsymbol{\beta})+\mathrm{V}(\mathrm{Z(s)})}$ using $V(\cdot)$ to denote the variance over the K spatial units, $\boldsymbol{\beta}$ is the vector of intercept and covariates, $\boldsymbol{X}$ the design matrix and Z(s) the Gaussian field

Table S4. Median and 95% credibility regions of the posterior of risk ratios from spatial regression analysis using a **log-Gaussian Cox process model for residence at diagnosis**.

covariates	All cancers		Leukaemia		Lymphoma		CNS tumours	
	Univariable[7]	Adjusted	Univariable[7]	Adjusted	Univariable[7]	Adjusted	Univariable[7]	Adjusted
NO_2[1]	1.03(1.00, 1.05)	1.02(0.99, 1.06)	1.03(0.99, 1.08)	1.05(0.99, 1.11)	1.07(1.00, 1.15)	1.04(0.95, 1.13)	1.01(0.95, 1.07)	1.00(0.93, 1.08)
Background radiation[2]	1.09(1.00, 1.18)	1.08(0.99, 1.18)	1.05(0.93, 1.19)	1.05(0.91, 1.21)	1.01(0.82, 1.23)	1.05(0.83, 1.30)	1.15(0.98, 1.35)	1.17(0.98, 1.40)
SEP[3]	1.02(0.99, 1.04)	1.01(0.98, 1.04)	0.99(0.95, 1.04)	0.98(0.93, 1.03)	1.03(0.96, 1.11)	1.02(0.94, 1.10)	1.06(1.00, 1.12)	1.06(1.00, 1.13)
YoR[4]	1.07(1.04, 1.1)	1.06(1.03, 1.09)	1.05(1.00, 1.10)	1.06(1.01, 1.11)	1.07(1.00, 1.15)	1.03(0.95, 1.12)	1.06(1.00, 1.14)	1.04(0.97, 1.12)
German[5]	1.00	1.00	1.00	1.00	1.00	1.00	1.00	1.00
French[5]	1.08(0.97, 1.19)	1.02(0.9, 1.15)	0.94(0.83, 1.07)	0.88(0.77, 1.01)	1.21(0.99, 1.47)	1.18(0.96, 1.44)	1.11(0.91, 1.35)	1.10(0.88, 1.36)
Italian[5]	1.07(0.88, 1.29)	0.97(0.78, 1.21)	1.01(0.78, 1.29)	0.90(0.66, 1.20)	0.97(0.63, 1.44)	0.89(0.55, 1.42)	1.12(0.77, 1.61)	0.98(0.64, 1.49)
Rural[6]	1.00	1.00	1.00	1.00	1.00	1.00	1.00	1.00
semi-urban[6]	1.04(0.96, 1.14)	1.02(0.93, 1.11)	1.04(0.90, 1.20)	1.02(0.88, 1.19)	1.01(0.80, 1.28)	0.97(0.76, 1.24)	1.05(0.88, 1.26)	1.00(0.83, 1.21)
urban[6]	1.04(0.97, 1.13)	0.99(0.90, 1.08)	1.00(0.88, 1.14)	0.93(0.80, 1.09)	1.19(0.98, 1.47)	1.10(0.86, 1.41)	1.05(0.90, 1.24)	0.99(0.81, 1.20)

Abbreviations: CNS: Central Nervous System, NO_2: Nitrogen Dioxide, SEP: Socio-Economic Position

[1] Modelled ambient Nitrogen Dioxide air concentration in $[\frac{\mu g}{m^3} \times 1000]$ and the interpretation of the term is per standard deviation of NO_2, ie per $77.7\frac{\mu g}{m^3} \times 1000$ (Figure S3)

[2] Modelled exposure to background ionising radiation in $[nSv/h]$ and the interpretation of the term is per standard deviation of radiation, ie per 60.2 nSv/h (Figure S4)

[3] Swiss socio-economical position, an index taking values from 0 to 100, with lower values indicating higher deprivation and the interpretation of the fixed effect is per standard deviation of SEP i.e. per 8.7 units (Figure S5)

[4] Duration in years of general cancer registration in the canton and the interpretation of the term is per standard deviation of the years i.e. per 11.6 years (Figure S6)

[5] Language speaking region as category with the baseline being the German speaking part of Switzerland (Figure S7)

[6] Levels of urbanisation as categorical with values rural, semi-urban and urban areas (Figure S8)

[7] These models are adjusted for age and year of diagnosis and include a spatial latent field as described in the main manuscript and the corresponding covariates.

Table S5. Median and 95% credibility regions of the posterior of risk ratios from spatial regression analysis using **log-Gaussian Cox process model for residence at birth**.

covariates	All cancers		Leukaemia		Lymphoma		CNS tumours	
	Univariable[7]	Adjusted	Univariable[7]	Adjusted	Univariable[7]	Adjusted	Univariable[7]	Adjusted
NO_2[1]	0.98(0.95, 1.01)	0.98(0.94, 1.02)	1.02(0.97, 1.07)	1.01(0.94, 1.08)	1.06(0.97, 1.16)	1.04(0.93, 1.17)	1.00(0.93, 1.06)	1.03(0.94, 1.12)
Background radiation[2]	1.13(1.04, 1.24)	1.14(1.03, 1.25)	1.03(0.89, 1.18)	1.01(0.85, 1.19)	1.09(0.85, 1.38)	1.20(0.89, 1.57)	1.12(0.94, 1.32)	1.13(0.91, 1.38)
SEP[3]	1.00(0.97, 1.03)	1.01(0.98, 1.04)	0.97(0.92, 1.03)	0.96(0.90, 1.01)	1.05(0.95, 1.15)	1.05(0.95, 1.16)	1.04(0.97, 1.11)	1.07(1.00, 1.16)
YoR[4]	1.02(0.99, 1.06)	1.02(0.98, 1.06)	1.06(1.01, 1.12)	1.08(1.02, 1.15)	1.01(0.92, 1.11)	0.96(0.86, 1.06)	0.98(0.91, 1.05)	0.96(0.88, 1.03)
German[5]	1.00	1.00	1.00	1.00	1.00	1.00	1.00	1.00
French[5]	1.09(0.98, 1.21)	1.08(0.95, 1.23)	1.02(0.88, 1.17)	0.94(0.79, 1.11)	1.23(0.96, 1.56)	1.29(0.98, 1.69)	1.08(0.89, 1.30)	1.15(0.93, 1.43)
Italian[5]	1.08(0.89, 1.31)	1.00(0.79, 1.26)	1.00(0.73, 1.33)	0.94(0.65, 1.34)	0.97(0.55, 1.60)	0.81(0.42, 1.49)	1.26(0.87, 1.79)	1.19(0.76, 1.82)
Rural[6]	1.00	1.00	1.00	1.00	1.00	1.00	1.00	1.00
semi-urban[6]	0.93(0.85, 1.03)	0.93(0.84, 1.03)	0.95(0.80, 1.14)	0.96(0.80, 1.15)	0.98(0.72, 1.34)	0.94(0.68, 1.30)	0.94(0.76, 1.15)	0.88(0.71, 1.10)
urban[6]	0.94(0.86, 1.02)	0.95(0.85, 1.06)	1.02(0.88, 1.18)	1.02(0.85, 1.23)	1.15(0.89, 1.50)	1.06(0.77, 1.47)	0.91(0.76, 1.09)	0.83(0.66, 1.04)

Abbreviations: CNS: Central Nervous System, NO_2: Nitrogen Dioxide, SEP: Socio-Economic Position

[1] Modelled ambient Nitrogen Dioxide air concentration in $[\frac{\mu g}{m^3} \times 1000]$ and the interpretation of the term is per standard deviation of NO_2, ie per $77.7\frac{\mu g}{m^3} \times 1000$ (Figure S3)

[2] Modelled exposure to background ionising radiation in $[nSv/h]$ and the interpretation of the term is per standard deviation of radiation, ie per 60.2 nSv/h (Figure S4)

[3] Swiss socio-economical position, an index taking values from 0 to 100, with lower values indicating higher deprivation and the interpretation of the fixed effect is per standard deviation of SEP i.e. per 8.7 units (Figure S5)

[4] Duration in years of general cancer registration in the canton and the interpretation of the term is per standard deviation of the years i.e. per 11.6 years (Figure S6)

[5] Language speaking region as category with the baseline being the German speaking part of Switzerland (Figure S7)

[6] Levels of urbanisation as categorical with values rural, semi-urban and urban areas (Figure S8)

[7] These models are adjusted for age and year of diagnosis and include a spatial latent field as described in the main manuscript and the corresponding covariates.

Table S6. Median and 95% credibility regions of the posterior of risk ratios from spatial regression using a **Besag-York-Mollié model for residence at diagnosis**.

covariates	All cancers		Leukaemia		Lymphoma		CNS tumours	
	Univariable[7]	Adjusted	Univariable[7]	Adjusted	Univariable[7]	Adjusted	Univariable[7]	Adjusted
NO_2[1]	1.02(0.99, 1.05)	1.02(0.99, 1.06)	1.04(1.00, 1.08)	1.06(1.00, 1.13)	1.09(1.02, 1.16)	1.07(0.98, 1.16)	1.00(0.94, 1.06)	0.98(0.91, 1.06)
Background radiation[2]	1.05(1.00, 1.10)	1.04(0.99, 1.09)	1.01(0.94, 1.07)	1.02(0.93, 1.11)	0.97(0.87, 1.08)	1.01(0.88, 1.15)	1.09(0.99, 1.20)	1.07(0.96, 1.20)
SEP[3]	1.02(0.98, 1.06)	1.00(0.96, 1.04)	1.01(0.96, 1.07)	0.96(0.89, 1.03)	1.04(0.95, 1.13)	0.98(0.87, 1.10)	1.03(0.95, 1.11)	1.02(0.93, 1.12)
YoR[4]	1.08(1.04, 1.11)	1.07(1.03, 1.11)	1.06(1.01, 1.11)	1.07(1.01, 1.13)	1.08(1.00, 1.16)	1.03(0.95, 1.12)	1.09(1.02, 1.17)	1.07(0.99, 1.16)
German[5]	1.00	1.00	1.00	1.00	1.00	1.00	1.00	1.00
French[5]	1.08(0.95, 1.22)	1.03(0.92, 1.15)	0.95(0.83, 1.08)	0.89(0.77, 1.02)	1.22(1.02, 1.46)	1.19(0.97, 1.44)	1.14(0.90, 1.44)	1.06(0.82, 1.37)
Italian[5]	1.11(0.87, 1.41)	1.00(0.80, 1.23)	1.00(0.77, 1.28)	0.91(0.67, 1.22)	0.93(0.60, 1.36)	0.87(0.53, 1.39)	1.21(0.77, 1.86)	1.09(0.67, 1.74)
Rural[6]	1.00	1.00	1.00	1.00	1.00	1.00	1.00	1.00
semi-urban[6]	1.06(0.97, 1.16)	1.04(0.95, 1.14)	1.07(0.92, 1.23)	1.05(0.89, 1.23)	0.99(0.78, 1.26)	0.95(0.74, 1.23)	1.11(0.92, 1.33)	1.09(0.89, 1.33)
urban[6]	1.07(0.99, 1.16)	1.03(0.93, 1.13)	1.04(0.91, 1.18)	0.96(0.81, 1.13)	1.21(0.99, 1.48)	1.09(0.84, 1.41)	1.11(0.94, 1.31)	1.10(0.89, 1.35)

Abbreviations: CNS: Central Nervous System, NO_2: Nitrogen Dioxide, SEP: Socio-Economic Position

[1] Modelled ambient Nitrogen Dioxide air concentration in $[\frac{\mu g}{m^3} \times 1000]$ and the interpretation of the term is per standard deviation of NO_2, ie per $60.9\frac{\mu g}{m^3} \times 1000$ (Figure S3)

[2] Modelled exposure to background ionising radiation in $[nSv/h]$ and the interpretation of the term is per standard deviation of radiation, ie per 37.5 nSv/h (Figure S4)

[3] Swiss socio-economical position, an index taking values from 0 to 100, with lower values indicating higher deprivation and the interpretation of the fixed effect is per standard deviation of SEP i.e. per 8.3 units (Figure S5)

[4] Duration in years of general cancer registration in the canton and the interpretation of the term is per standard deviation of the years i.e. per 12.4 years (Figure S6)

[5] Language speaking region as category with the baseline being the German speaking part of Switzerland (Figure S7)

[6] Levels of urbanisation as categorical with values rural, semi-urban and urban areas (Figure S8)

[7] These models are adjusted for age and year of diagnosis and include a spatial latent field as described in Text 2 and the corresponding covariates.

Table S7. Median and 95% credibility regions of the posterior of risk ratios from spatial regression using **Besag-York-Mollié model for residence at birth**.

covariates	All cancers		Leukaemia		Lymphoma		CNS tumours	
	Univariable[7]	Adjusted	Univariable[7]	Adjusted	Univariable[7]	Adjusted	Univariable[7]	Adjusted
NO_2[1]	0.98(0.95, 1.01)	1.00(0.96, 1.04)	1.00(0.96, 1.05)	1.02(0.95, 1.10)	0.99(0.92, 1.08)	1.00(0.9, 1.12)	0.97(0.92, 1.03)	1.02(0.94, 1.11)
Background radiation[2]	1.06(1.01, 1.11)	1.06(1.00, 1.12)	1.01(0.94, 1.09)	0.99(0.89, 1.09)	1.06(0.93, 1.20)	1.11(0.93, 1.3)	1.06(0.97, 1.16)	1.05(0.92, 1.18)
SEP[3]	0.98(0.94, 1.02)	1.01(0.96, 1.07)	0.96(0.90, 1.03)	0.92(0.84, 1.01)	0.97(0.87, 1.09)	1.03(0.88, 1.20)	0.95(0.88, 1.03)	1.02(0.91, 1.14)
YoR[4]	1.02(0.99, 1.06)	1.01(0.97, 1.05)	1.05(0.99, 1.11)	1.08(1.01, 1.15)	0.98(0.89, 1.07)	0.94(0.84, 1.04)	0.97(0.91, 1.04)	0.97(0.90, 1.05)
German[5]	1.00	1.00	1.00	1.00	1.00	1.00	1.00	1.00
French[5]	1.09(0.99, 1.19)	1.08(0.96, 1.2)	1.00(0.87, 1.15)	0.91(0.76, 1.08)	1.18(0.95, 1.47)	1.26(0.97, 1.61)	1.04(0.88, 1.23)	1.08(0.89, 1.31)
Italian[5]	1.08(0.91, 1.29)	1.00(0.8, 1.23)	0.97(0.71, 1.28)	0.91(0.63, 1.29)	0.95(0.55, 1.52)	0.81(0.43, 1.44)	1.25(0.89, 1.70)	1.19(0.79, 1.77)
Rural[6]	1.00	1.00	1.00	1.00	1.00	1.00	1.00	1.00
semi-urban[6]	0.93(0.85, 1.03)	0.93(0.83, 1.03)	0.92(0.77, 1.09)	0.95(0.78, 1.15)	0.86(0.63, 1.17)	0.87(0.63, 1.22)	0.89(0.73, 1.10)	0.88(0.70, 1.11)
urban[6]	0.94(0.86, 1.02)	0.92(0.83, 1.03)	0.97(0.84, 1.12)	0.99(0.81, 1.20)	0.98(0.77, 1.28)	1.01(0.73, 1.41)	0.83(0.70, 0.99)	0.80(0.63, 1.01)

Abbreviations: CNS: Central Nervous System, NO_2: Nitrogen Dioxide, SEP: Socio-Economic Position

[1] Modelled ambient Nitrogen Dioxide air concentration in $[\frac{\mu g}{m^3}\times 1000]$ and the interpretation of the term is per standard deviation of NO_2, ie per $60.9\frac{\mu g}{m^3}\times 1000$ (Figure S3)

[2] Modelled exposure to background ionising radiation in $[nSv/h]$ and the interpretation of the term is per standard deviation of radiation, ie per 37.5 nSv/h (Figure S4)

[3] Swiss socio-economical position, an index taking values from 0 to 100, with lower values indicating higher deprivation and the interpretation of the fixed effect is per standard deviation of SEP i.e. per 8.3 units (Figure S5)

[4] Duration in years of general cancer registration in the canton and the interpretation of the term is per standard deviation of the years i.e. per 12.4 years (Figure S6)

[5] Language speaking region as category with the baseline being the German speaking part of Switzerland (Figure S7)

[6] Levels of urbanisation as categorical with values rural, semi-urban and urban areas (Figure S8)

[7] These models are adjusted for age and year of diagnosis and include a spatial latent field as described in Text 2 and the corresponding covariates.

Figure S1. Expected number of cancers cases E_k adjusted by age group (0-4, 5-9, 10-15) and year of diagnosis per municipality, see Text S1 for definition.

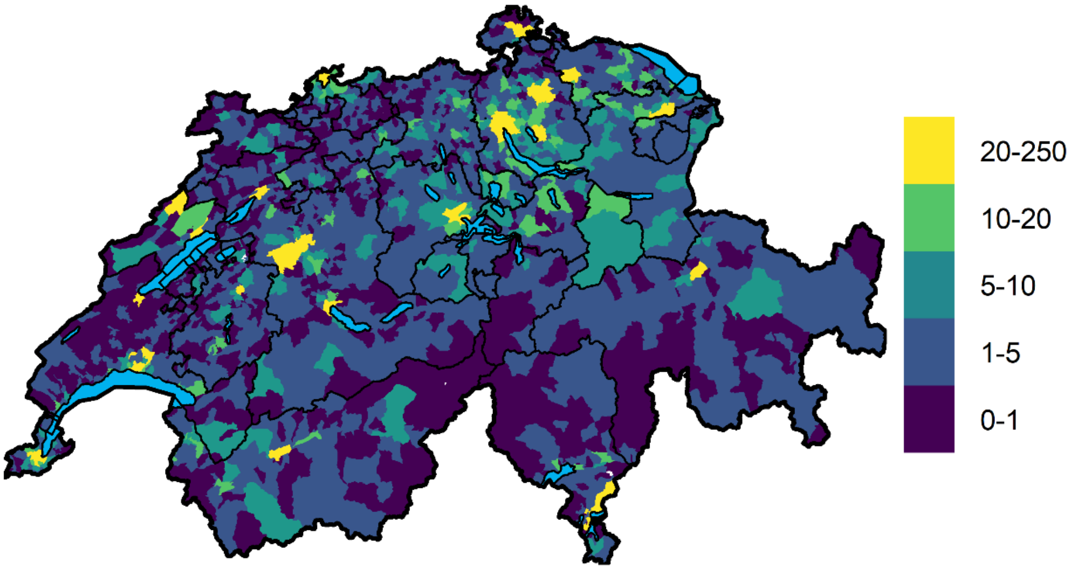

Figure S2. Expected number of cancers cases E_k adjusted by age group (0-4, 5-9, 10-15) and year of diagnosis per $1km \times 1km$ grid cell, see Text S1 for definition.

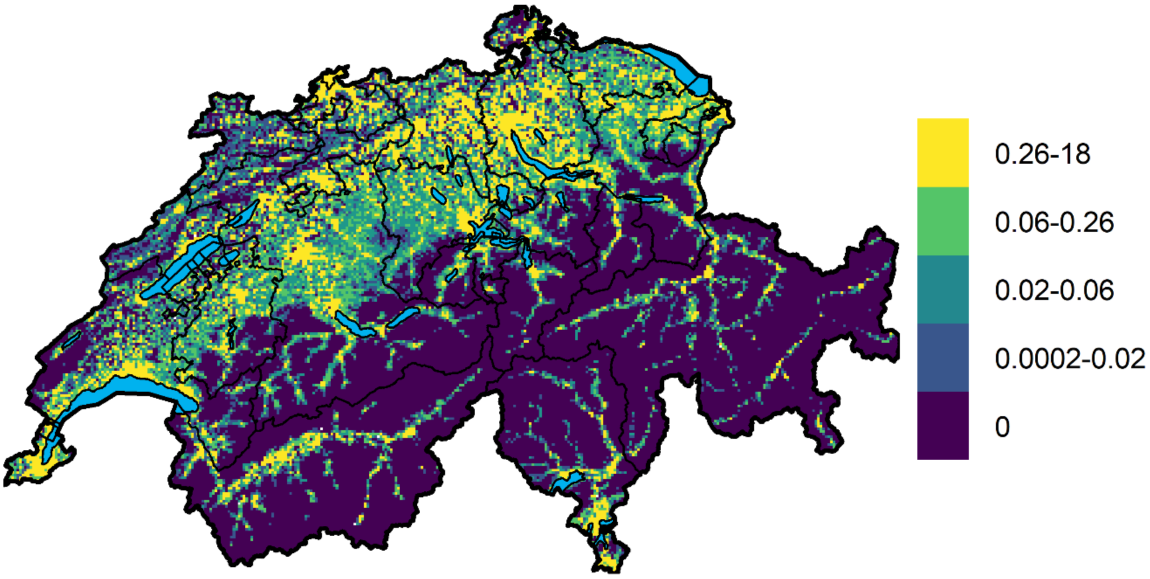

Figure S3. Modelled ambient NO_2 [$\mu g/m^3 \times 1000$] air concentration per municipality and on a 500x500 grid. The mean is taken over 1990, 2000 and 2010. The municipality values were calculated by taking the mean of the values of the 200x200 grid cells whose centroids fall into the corresponding municipality. Data was made available after request to Meteotest (www.meteotest.ch).

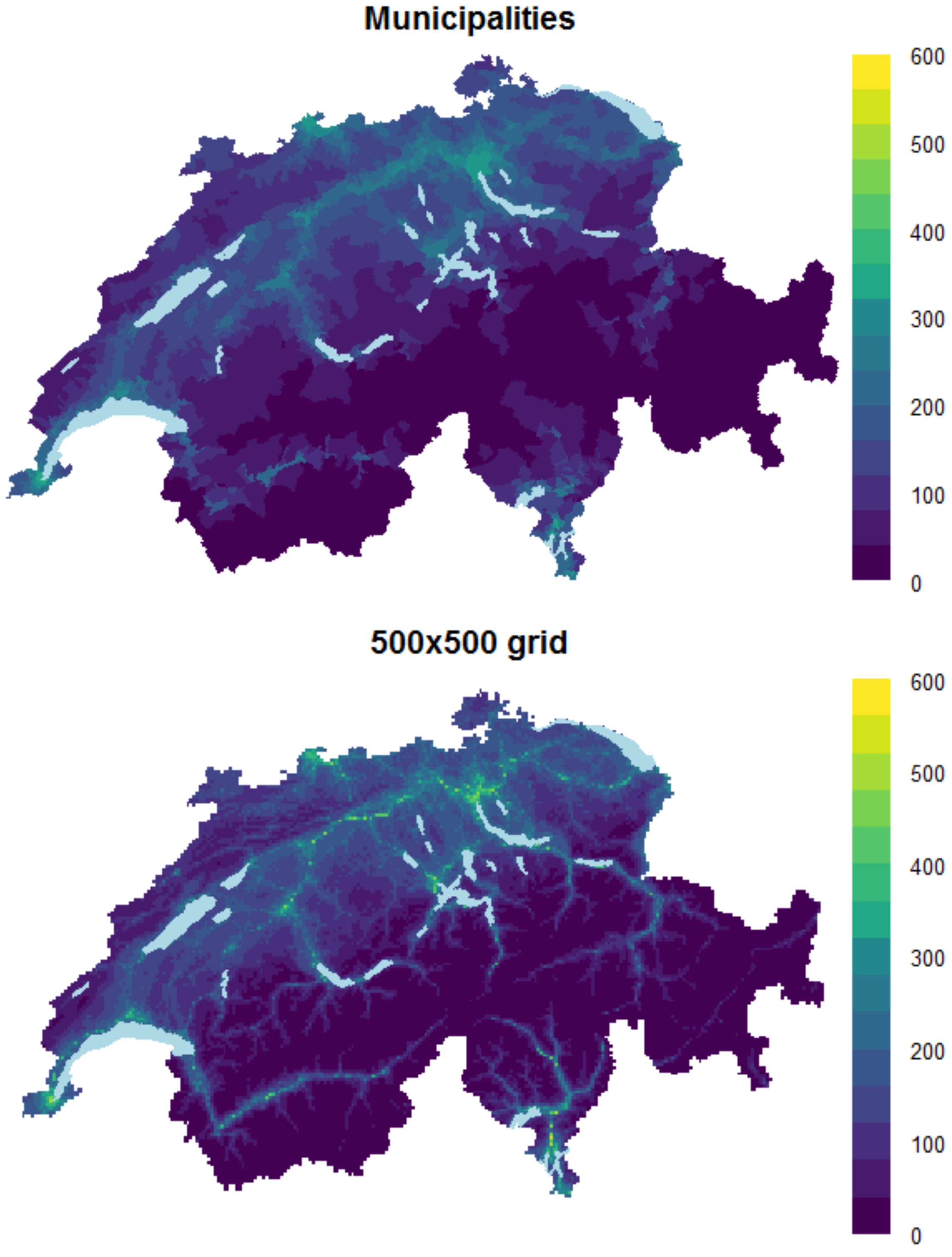

Figure S4. Modelled exposure to background ionising radiation [nSv/h] from cosmic and terrestrial radiation per municipality and on a $2 \times 2km^2$ grid. [6, 7] The municipality values were calculated by taking the mean of the values of the $2 \times 2km^2$ grid cells whose centroids fall into the corresponding municipality.

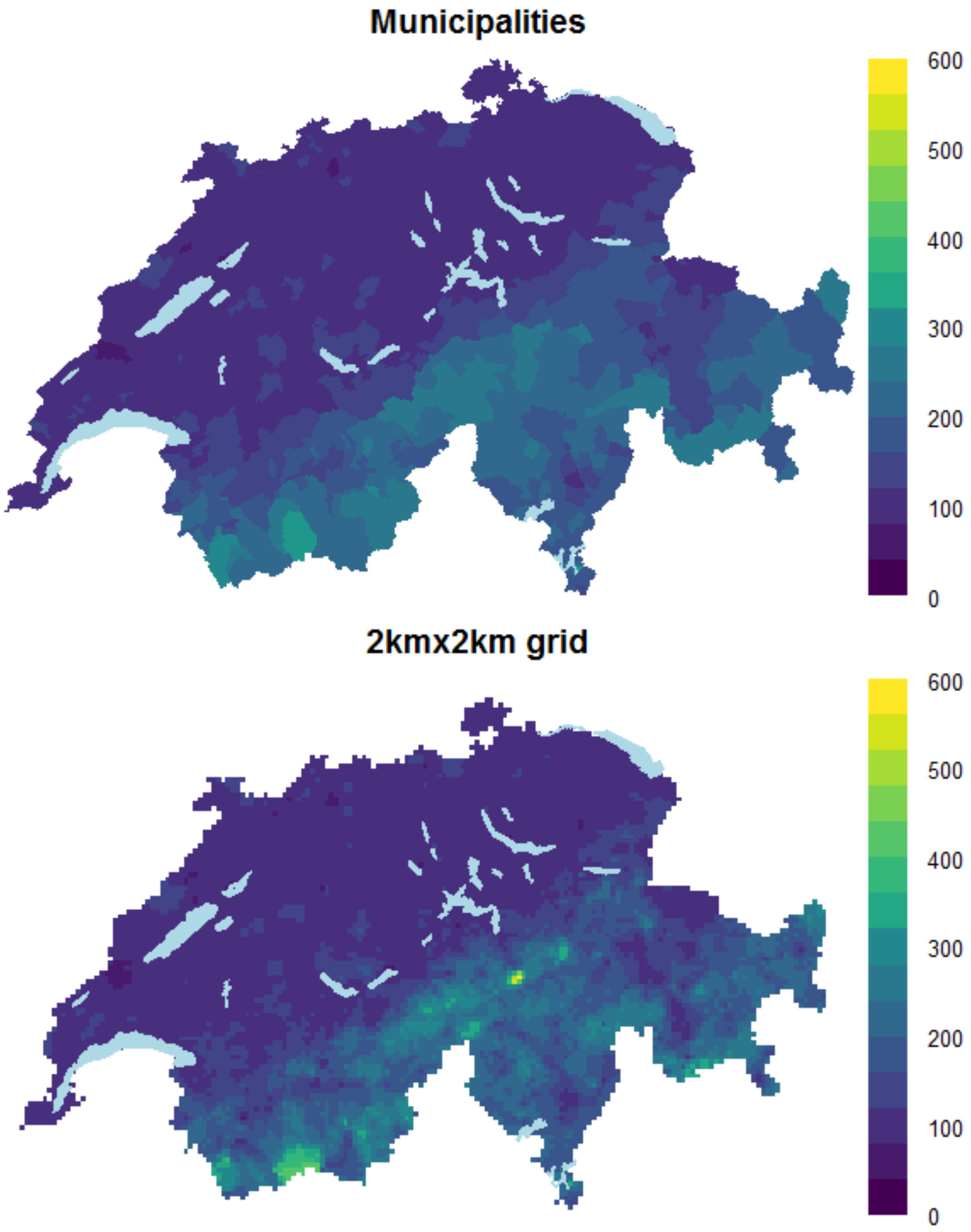

Figure S5. Swiss socioeconomic (SEP) index as mean per municipality and a combination of municipality mean and 500x500m^2 grid means. [8] The mean was taken as over the buildings inside the corresponding municipality or 500x500m^2 grid cell. Higher values indicate more affluent groups.

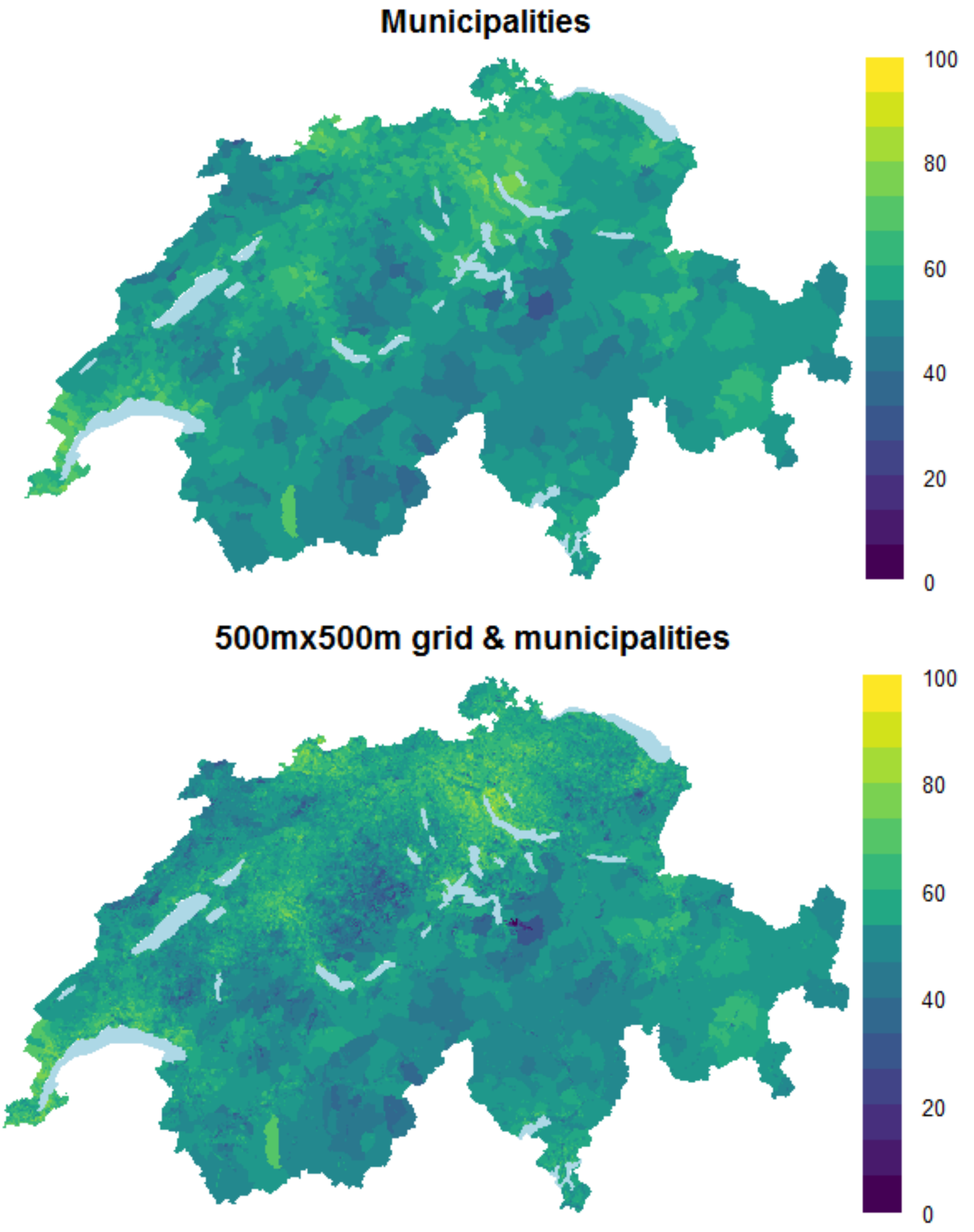

Figure S6. Duration in years of general cancer registration in the canton for period 1985-2015. The data is freely available from the Swiss National Institution for Cancer Epidemiology and Registration (http://www.nicer.org/).

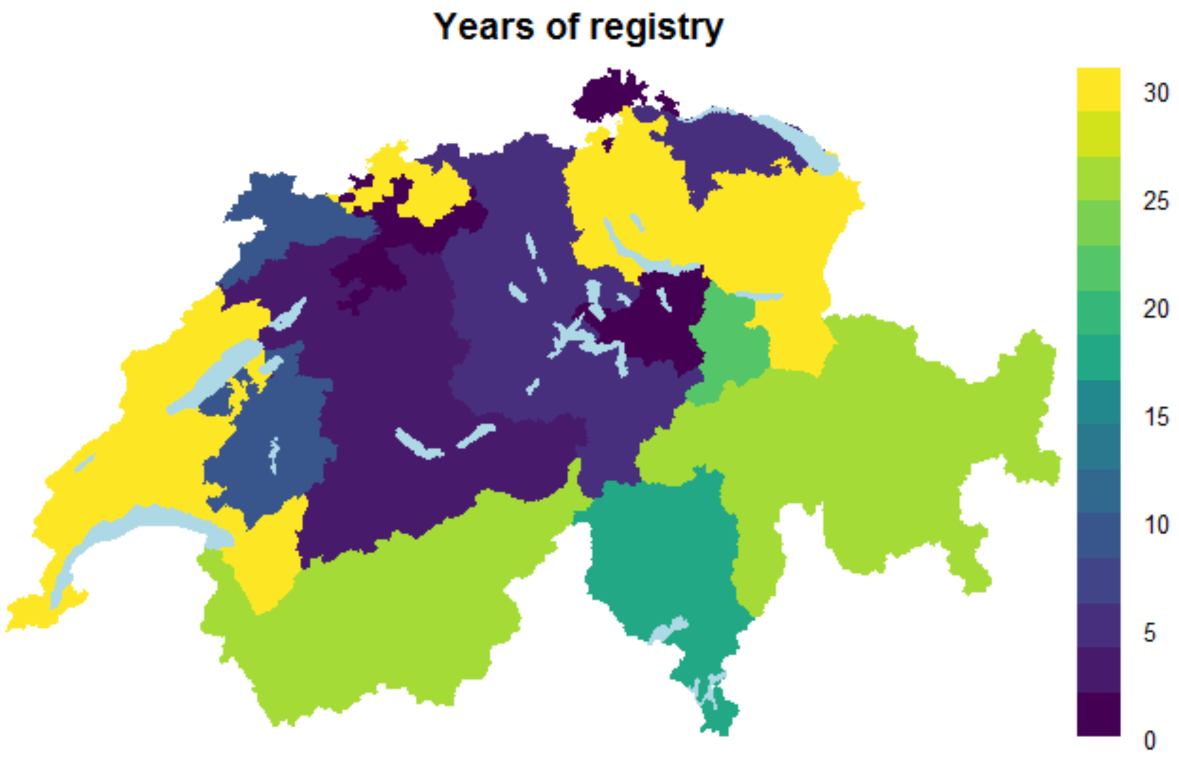

Figure S7. Language regions per municipality in Switzerland. Data is freely available from the Federal Office of Statistics (https://www.bfs.admin.ch/bfs/de/home/grundlagen/raumgliederungen.html). In the main analysis, the Raetoromanisch region was grouped with German speaking region.

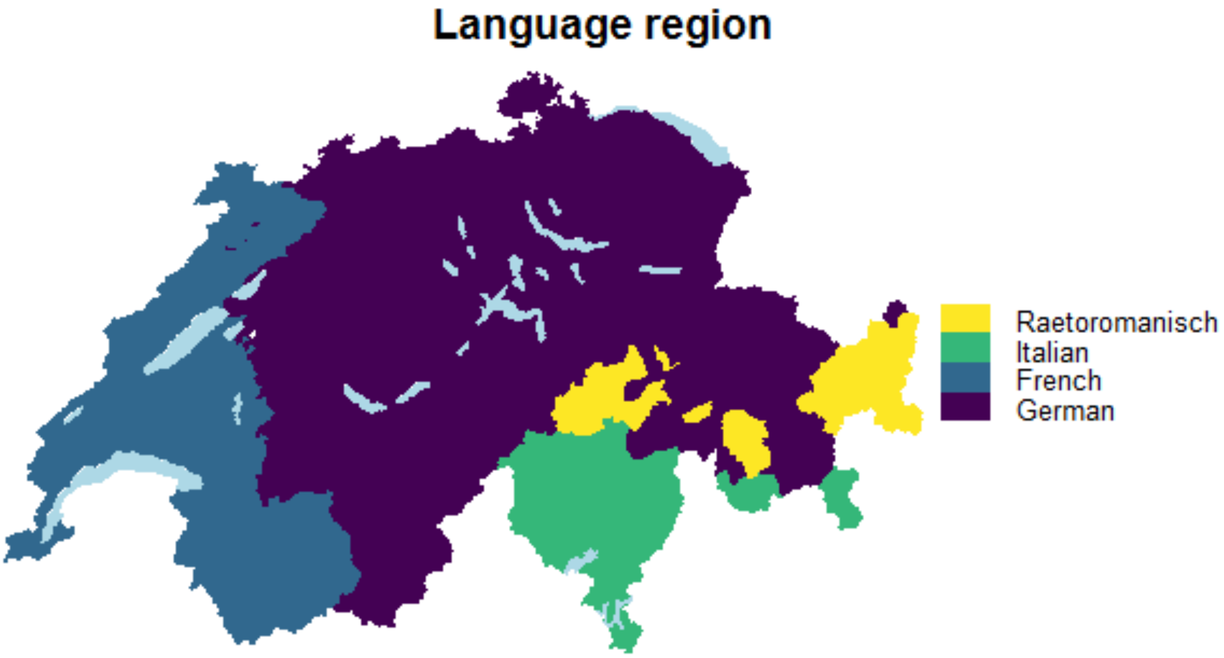

Figure S8. Levels of urbanisation in Switzerland categorized as urban, semi-rural and rural areas. Data is freely available from the Federal Office of Statistics (https://www.bfs.admin.ch/bfs/de/home/grundlagen/raumgliederungen.html).

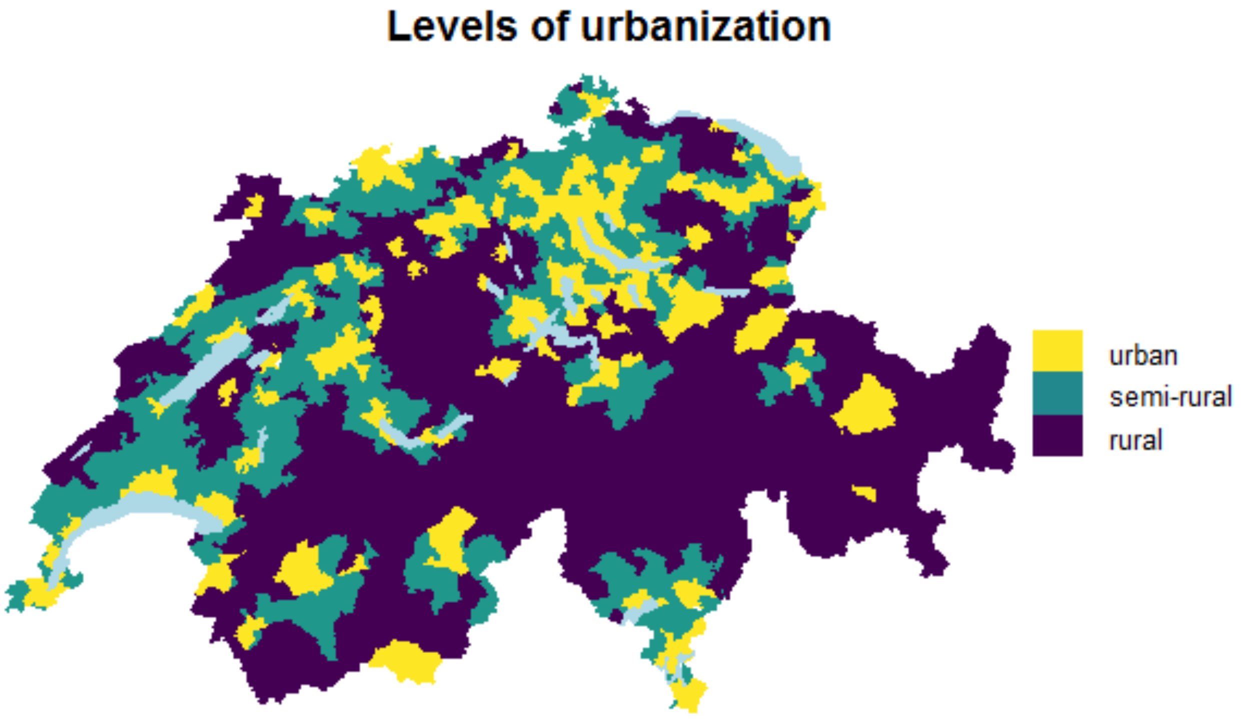

Figure S9. Autoregressive processes of order 1 to examine a more flexible fit for background ionising radiation, ambient NO_2, and socio-economic position. The AR(1) was added as an additional term in Equation 2.2.1 Text S2, but without the covariates. These results refer to all cancers combined using residence of diagnosis. The solid line is the pointwise posterior median, whereas the black dashed lines pointwise 95% credibility regions of the relative risk trends over the different values of the covariates.

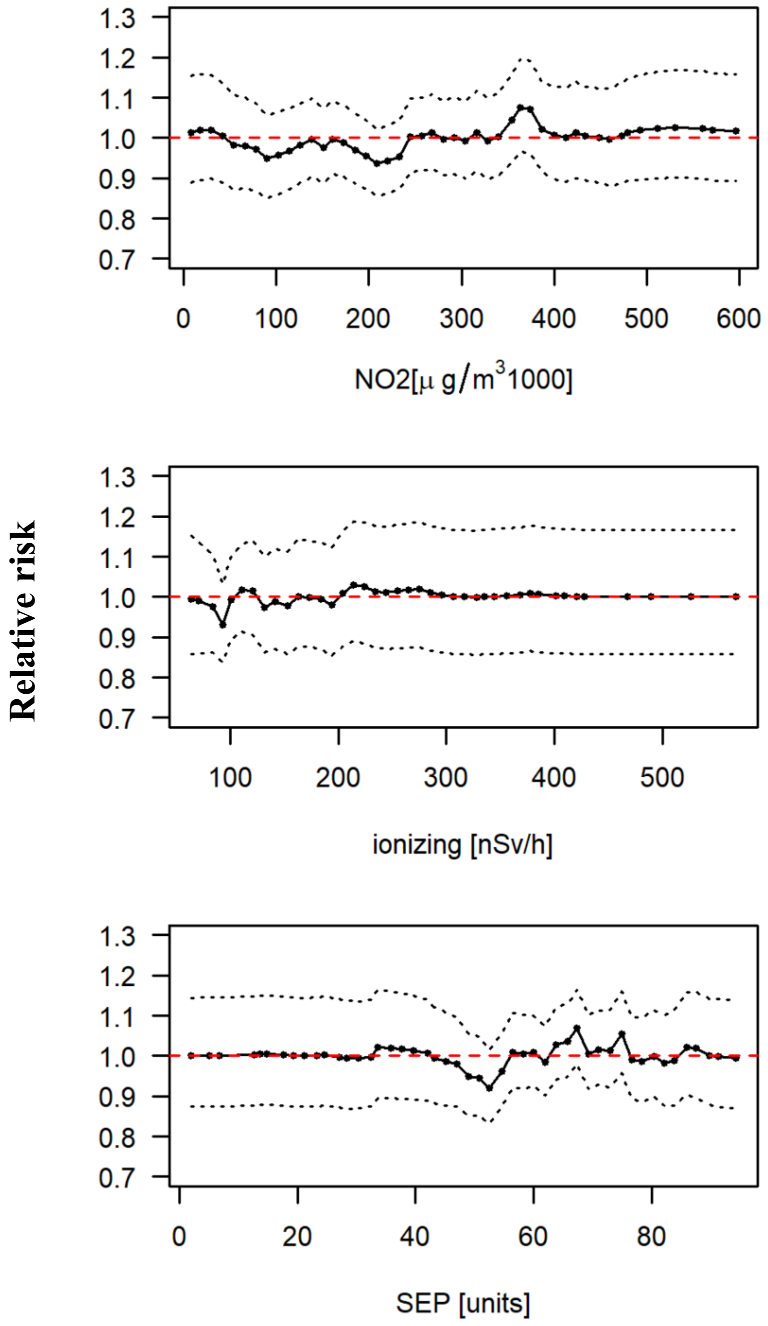

Abbreviations: SEP: socio-economic position

Figure S10. Modelled relative risk surfaces based on log-Gaussian Cox processes (LGCPs) and residence at birth: Maps of median posterior of grid specific relative risk during 1985-2015 of cancer incidence stratified by diagnostic group based. The left panel refers to the models adjusted for age and year of diagnosis, whereas the right panel to the fully adjusted models (adjusted for modelled ambient NO_2, modelled exposure to background ionising radiation, duration in years of general cancer registration in the canton, linguistic region and degree of urbanicity).

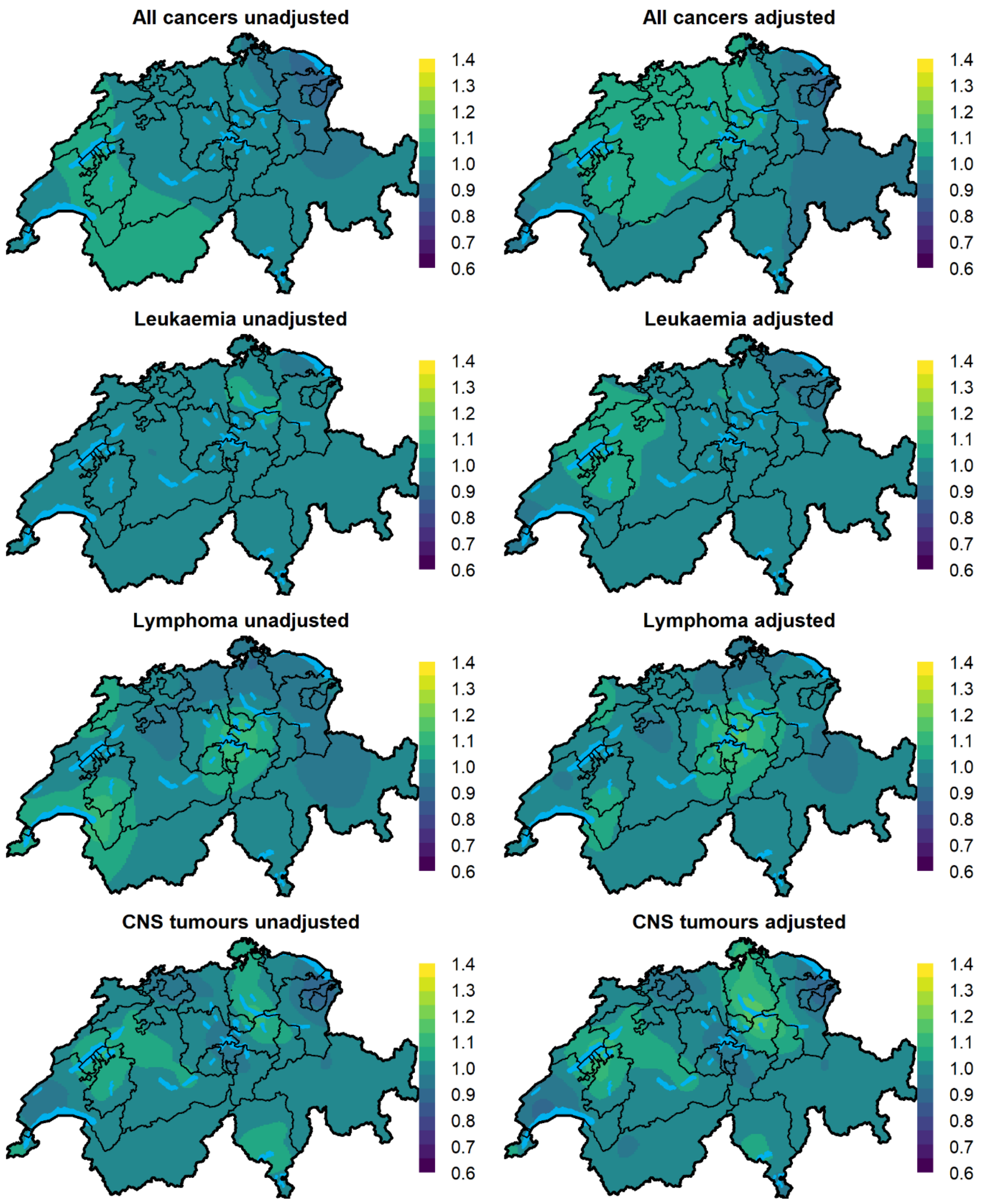

Figure S11. Exceedance probability surfaces based on log-Gaussian Cox processes (LGCPs) and residence at birth: Maps of exceedance probability based on log-Gaussian Cox processes and the place of birth. Exceedance probability was defined as the probability that the grid specific relative risk is larger than 1. The left panel refers to the models adjusted for age and year of diagnosis, whereas the right panel to the fully adjusted models (adjusted for modelled ambient NO_2, modelled exposure to background ionising radiation, duration in years of general cancer registration in the canton, linguistic region and degree of urbanicity).

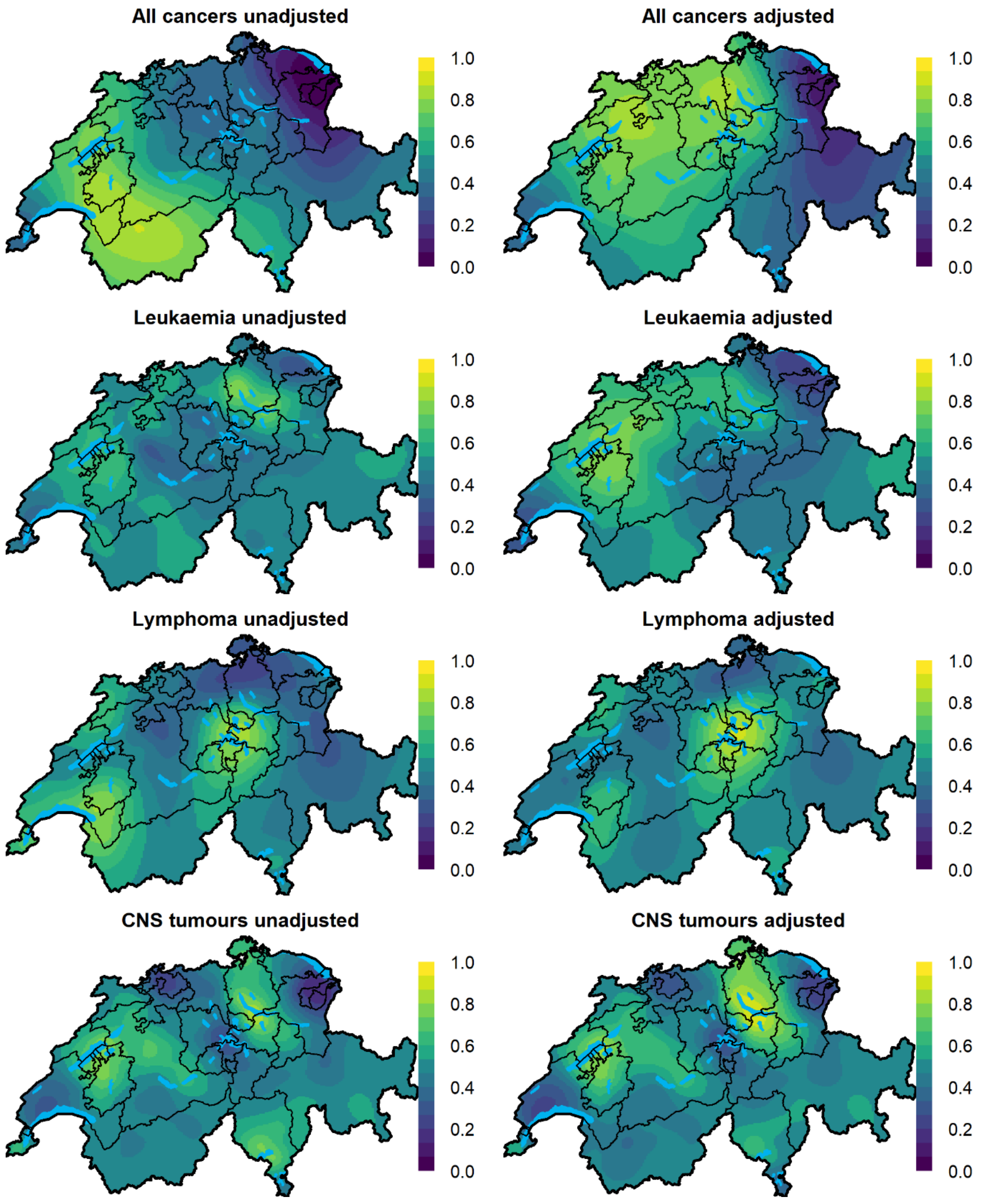

Figure S12. Modelled relative risk surfaces based on Besag-York-Mollié (BYM) and residence at diagnosis: Maps of median posterior of grid specific relative risk during 1985-2015 of cancer incidence stratified by diagnostic group based on the BYM model and the residence of diagnosis. The left panel refers to the models adjusted for age and year of diagnosis, whereas the right panel to the fully adjusted models (adjusted for modelled ambient NO_2, modelled exposure to background ionising radiation, duration in years of general cancer registration in the canton, linguistic region and degree of urbanicity).

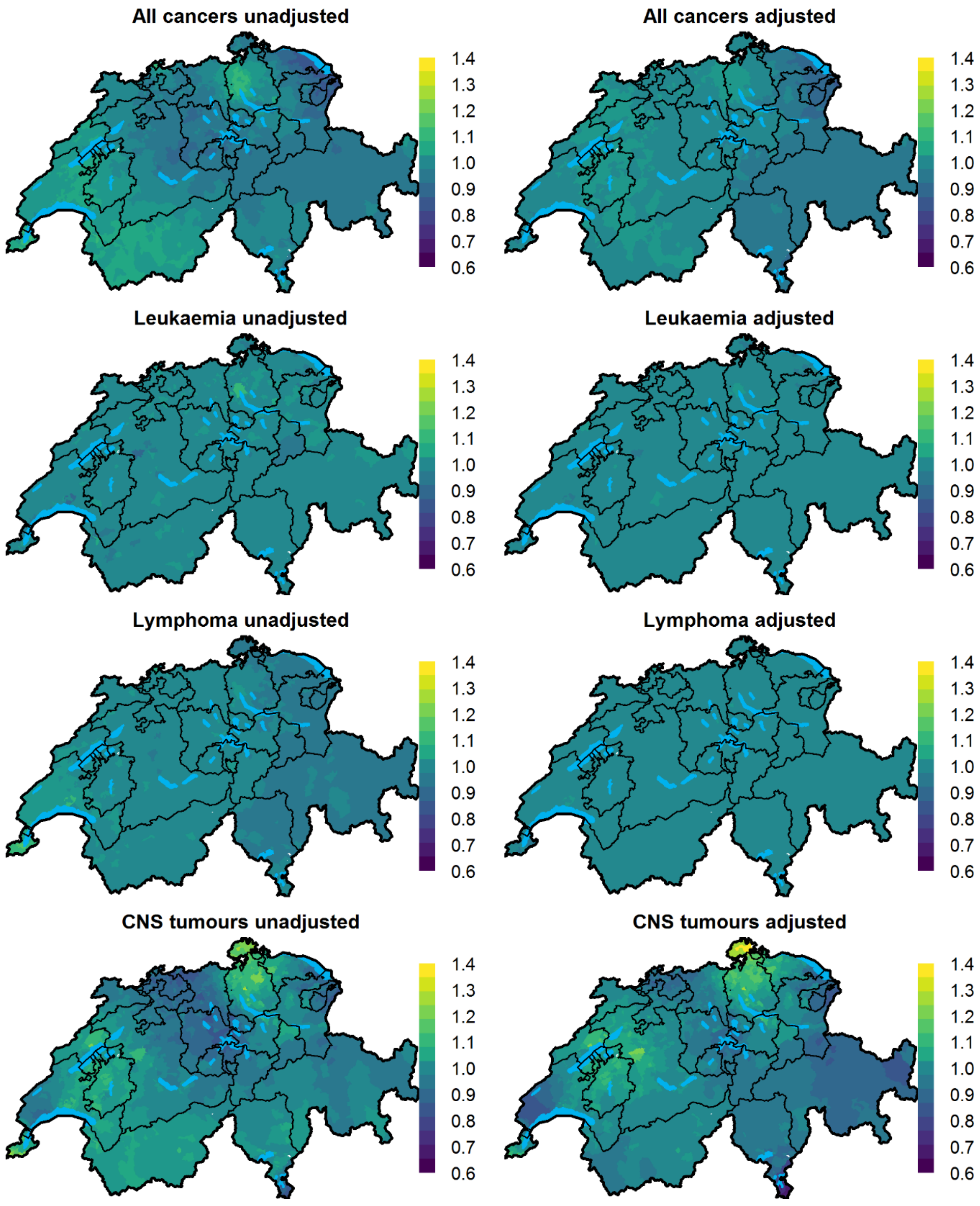

Figure S13. Exceedance probability surfaces based on Besag-York-Mollié (BYM) and residence at diagnosis: Maps of exceedance probability based on the BYM model and the residence of diagnosis. Exceedance probability was defined as the probability that the municipality specific relative risk is larger than 1. The left panel refers to the models adjusted for age and year of diagnosis, whereas the right panel to the fully adjusted models (adjusted for modelled ambient NO_2, modelled exposure to background ionising radiation, duration in years of general cancer registration in the canton, linguistic region and degree of urbanicity).

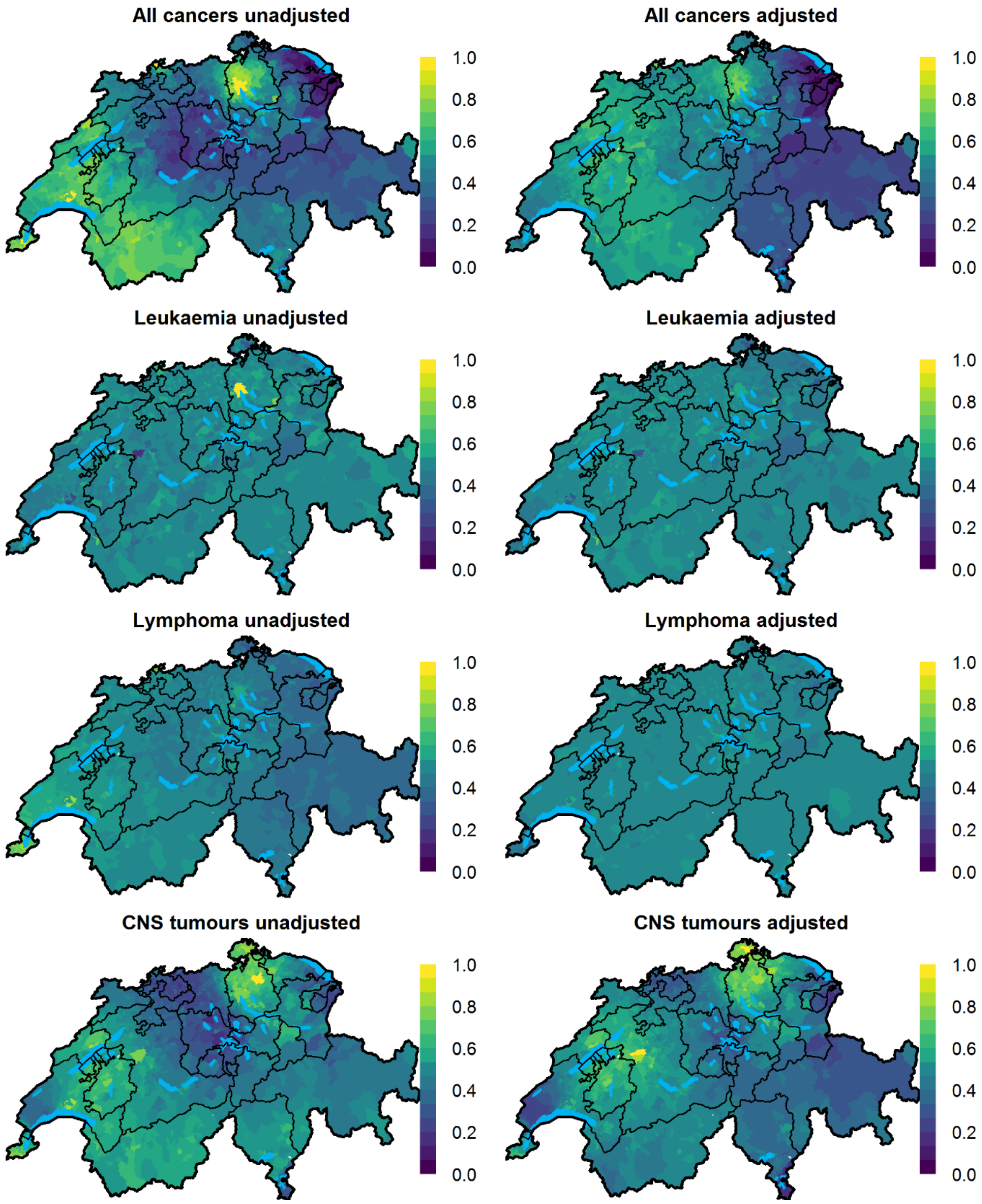

Figure S14. Modelled relative risk surfaces based on Besag-York-Mollié (BYM) and residence at birth: Maps of median posterior of grid specific relative risk during 1985-2015 of cancer incidence stratified by diagnostic group based on the BYM model and the place of birth. The left panel refers to the models adjusted for age and year of diagnosis, whereas the right panel to the fully adjusted models (adjusted for modelled ambient NO_2, modelled exposure to background ionising radiation, duration in years of general cancer registration in the canton, linguistic region and degree of urbanicity).

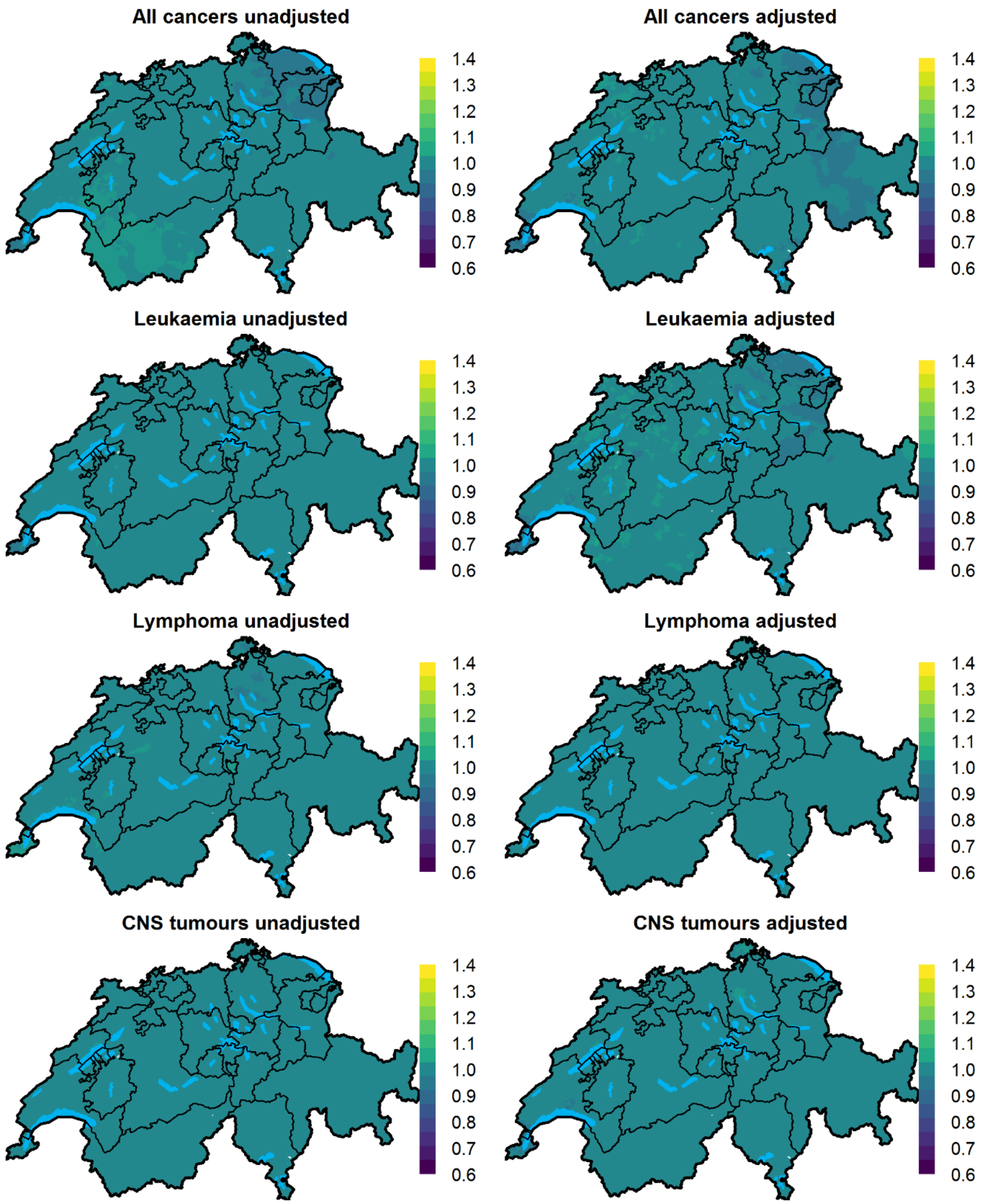

Figure S15. Exceedance probability surfaces based on Besag-York-Mollié (BYM) and residence at birth: Maps of exceedance probability based on the BYM model and the residence of birth. Exceedance probability was defined as the probability that the municipality specific relative risk is larger than 1. The left panel refers to the models adjusted for age and year of diagnosis, whereas the right panel to the fully adjusted models (adjusted for modelled ambient NO_2, modelled exposure to background ionising radiation, duration in years of general cancer registration in the canton, linguistic region and degree of urbanicity).

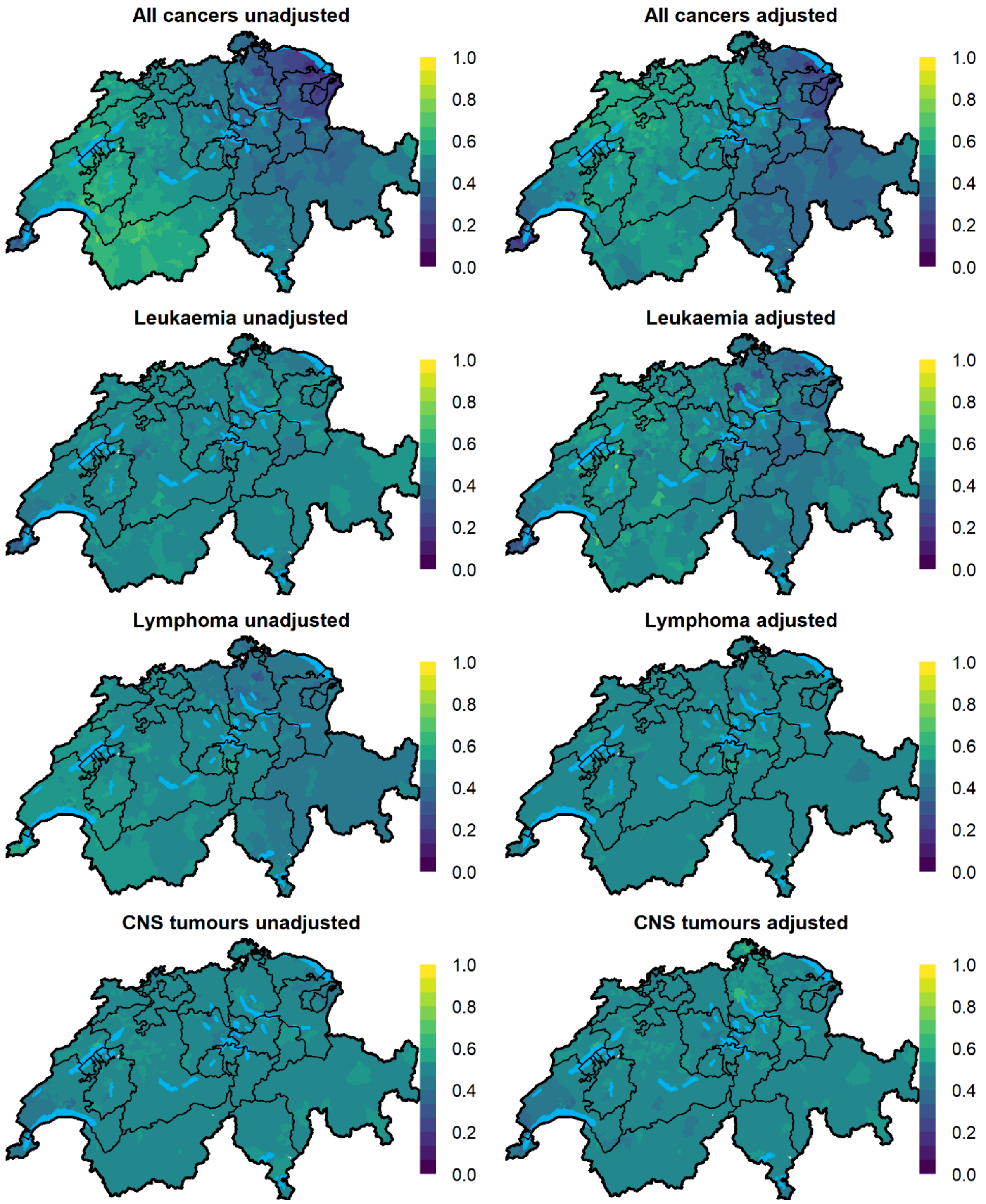

Figure S16. Sensitivity of relative risk surfaces of all cancers using log-Gaussian Cox processes, the unadjusted model and different priors for the range parameter: Sensitivity analysis of the posterior median of the grid specific relative risk using different penalized complexity priors for the range parameter and focusing on all childhood cancers combined and residence of diagnosis using the unadjusted model. Notice that for the model presented in the paper we used $\rho = 60km$.

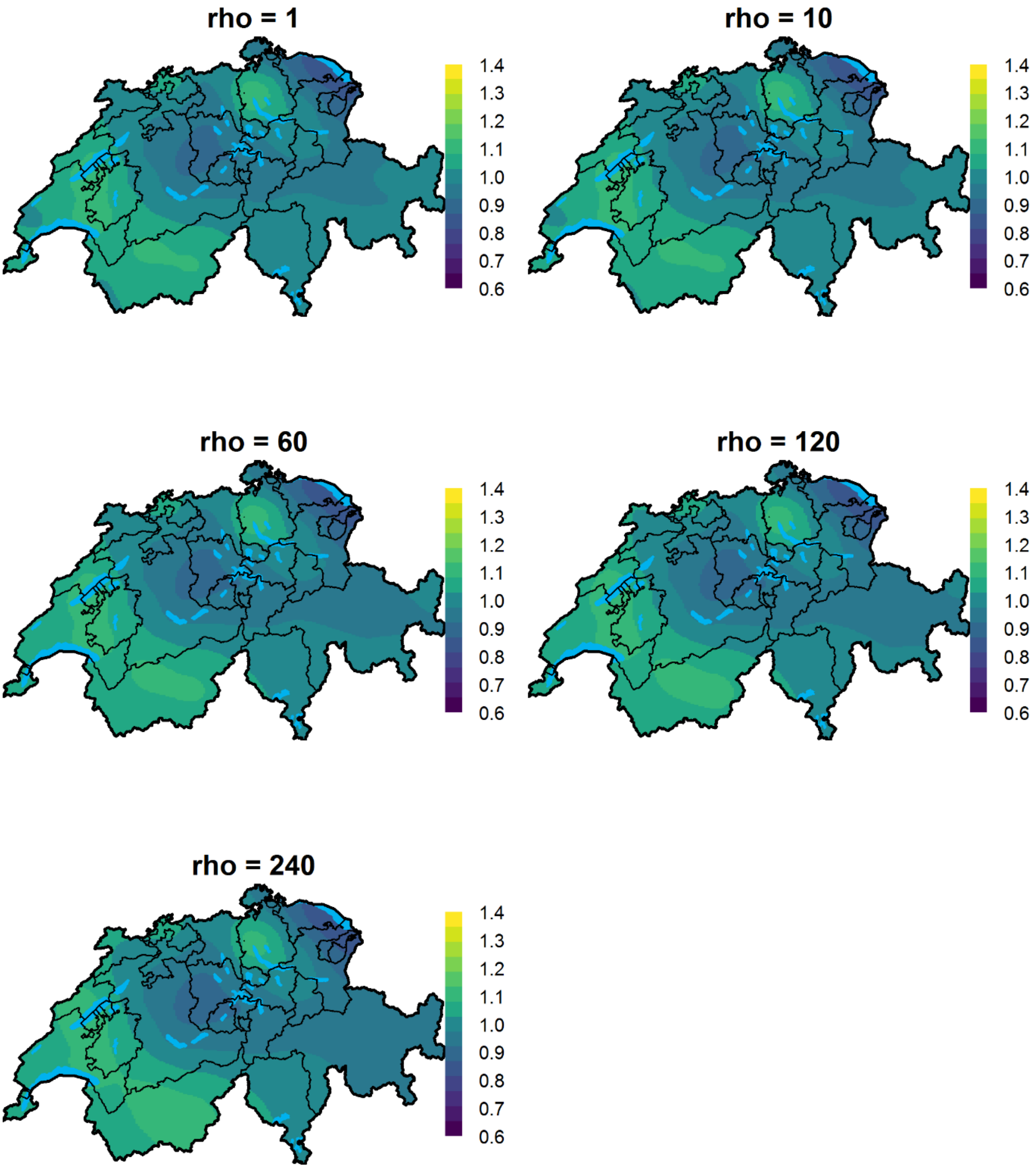

Figure S17. Boxplots of relative risk surfaces of all cancers using log-Gaussian Cox processes, the unadjusted model and different priors for the range parameter: Boxplots of the median posterior of grid specific relative risk using different penalized complexity (PC) priors for the range parameter (ρ) and focusing on all childhood cancers combined and place of diagnosis using the unadjusted model. Notice that for the model presented in the paper we used $\rho = 60m$.

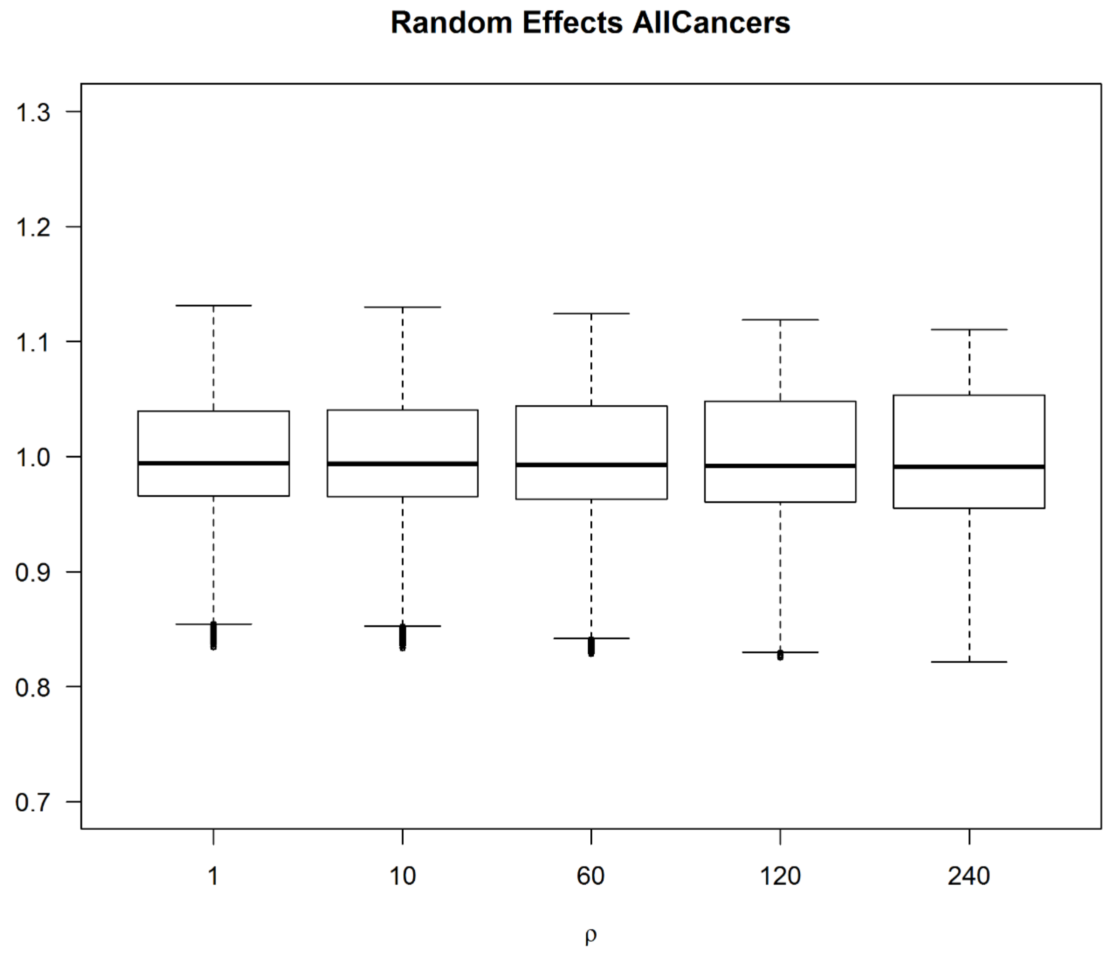

Figure S18. Sensitivity of relative risk surfaces of all cancers using log-Gaussian Cox processes, the fully adjusted model and different priors for the range parameter: Sensitivity analysis of the median posterior of grid specific relative risk using different penalized complexity (PC) priors for the range parameter (ρ) and focusing on all childhood cancers combined and place of diagnosis using the fully adjusted model (adjusted for modelled ambient NO_2, modelled exposure to background ionising radiation, duration in years of general cancer registration in the canton, linguistic region and degree of urbanicity). Notice that for the model presented in the paper we used $\rho = 60m$.

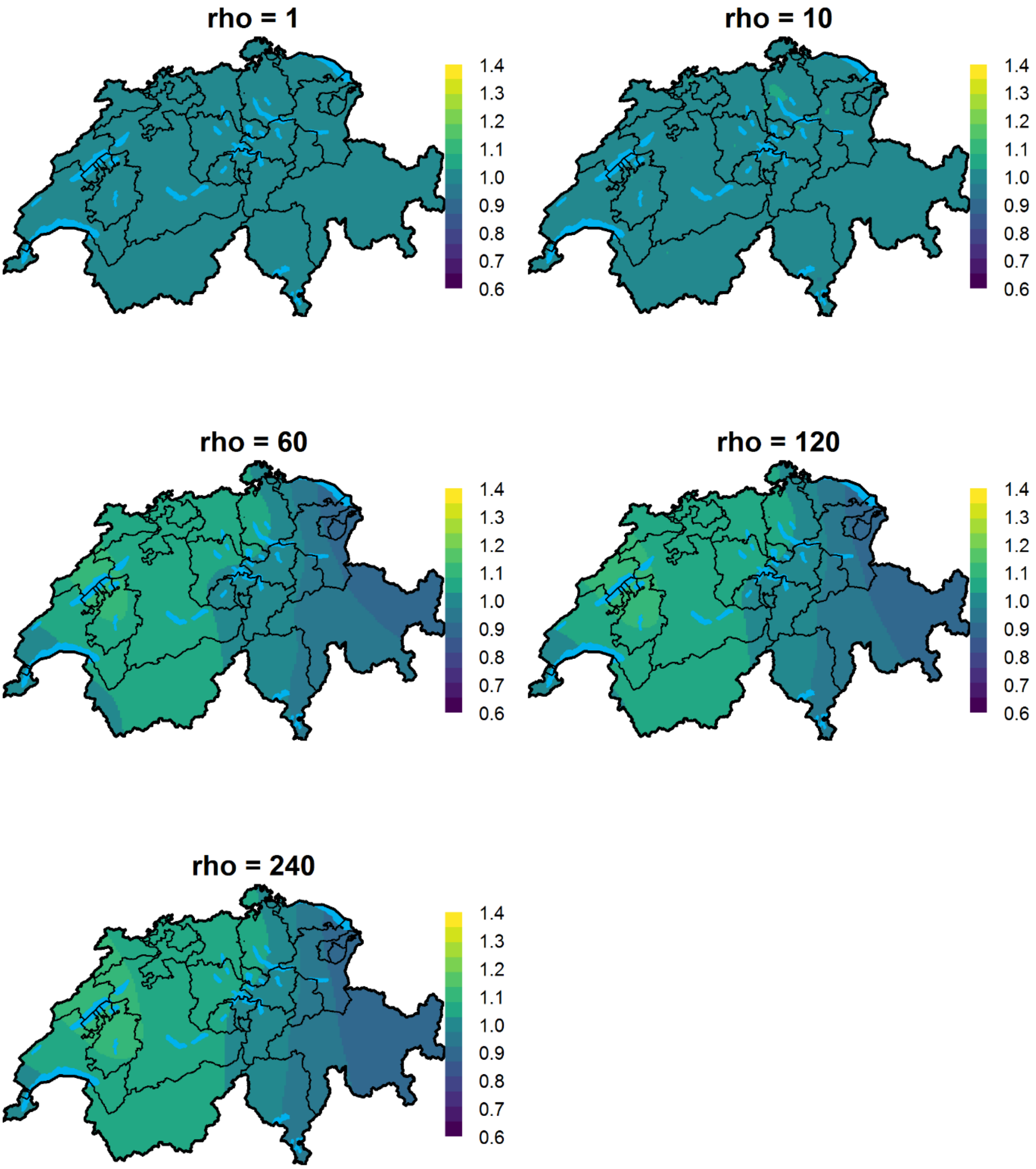

Figure S19. Boxplots of relative risk surfaces of all cancers using log-Gaussian Cox processes, the fully adjusted model and different priors for the range parameter: Boxplots of the median posterior of grid specific relative risk using different penalized complexity (PC) priors for the range parameter (ρ) and focusing on all childhood cancers combined and place of diagnosis using the fully adjusted model (adjusted for modelled ambient NO_2, modelled exposure to background ionising radiation, duration in years of general cancer registration in the canton, linguistic region and degree of urbanicity). Notice that for the model presented in the paper we used $\rho = 60m$.

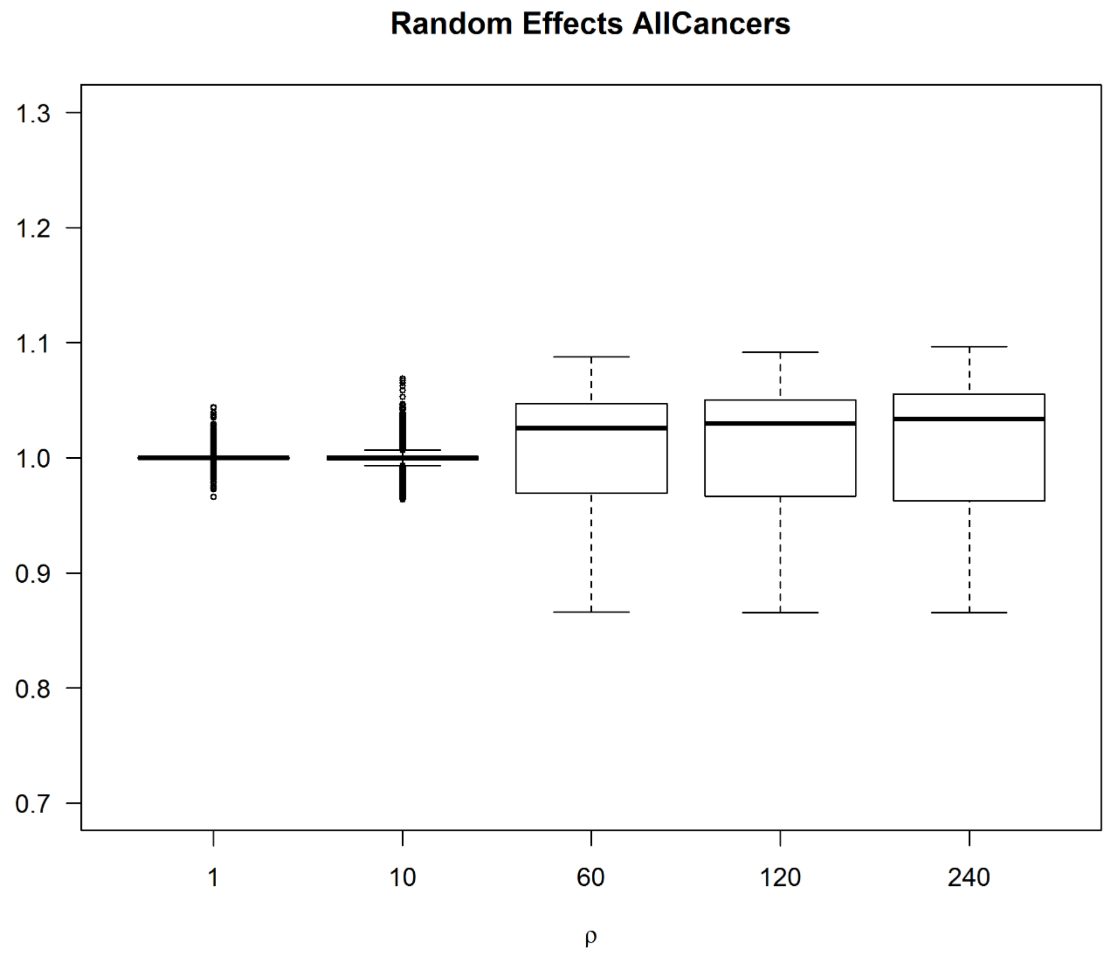

Figure S20. Regression coefficients of all cancers using log-Gaussian Cox processes, the fully adjusted model and different priors for the range parameter: Sensitivity analysis of the regression coefficients using different penalized complexity (PC) priors for the range parameter (ρ) and focusing on all childhood cancers combined and place of diagnosis using the fully adjusted model (adjusted for modelled ambient NO_2, modelled exposure to background ionising radiation, duration in years of general cancer registration in the canton, linguistic region and degree of urbanicity). Notice that for the model presented in the paper we used $\rho = 60m$.

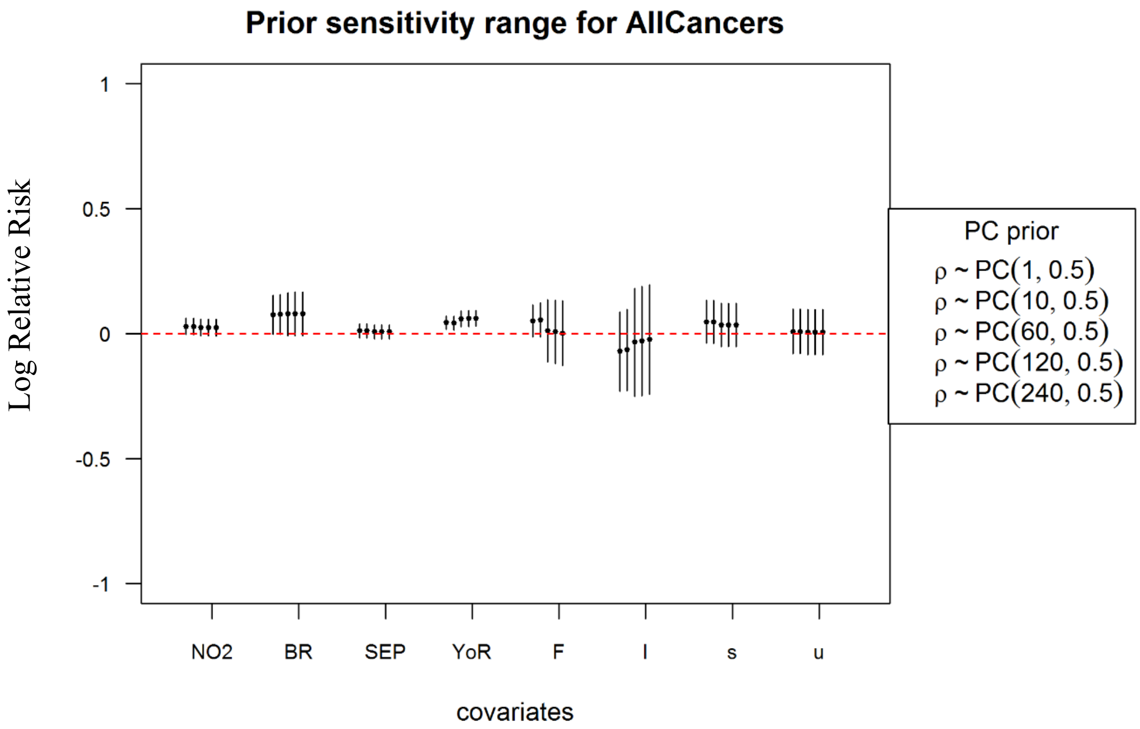

Abrevations: NO_2: Nitrogen Dioxide (Figure S3), BR: Total dose background radiation (Figure S4), SEP: Socio-Economic Position (Figure S5), YoR: years of existing cantonal registry (Figure S6), F: French speaking part, I: Italian speaking part (Figure S7), s: semi-urban areas, u: urban areas (Figure S8)

NO_2, background ionising radiation, SEP and years of cantonal registry were scaled and considered as linear effects. Their interpretation is a mulitplicative increase (or dicrease) in the number of observed cases compared to the number of the expected cases per 1sd increase (or dicrease) in the covariate. The sd for NO_2 is 77.7 $\mu g/m^3 \times 10$, for total background radiation 60.2 nSv/h, for SEP 8.7 units and for years of cantonal registry 11.6 years.

Figure S21. Sensitivity of relative risk surfaces of CNS tumours using log-Gaussian Cox processes, the unadjusted model and different priors for the range parameter: Sensitivity analysis of the median posterior of grid specific relative risk using different penalized complexity (PC) priors for the range parameter (ρ) and focusing on CNS tumours and place of diagnosis using the unadjusted model. Notice that for the model presented in the paper we used $\rho = 60m$.

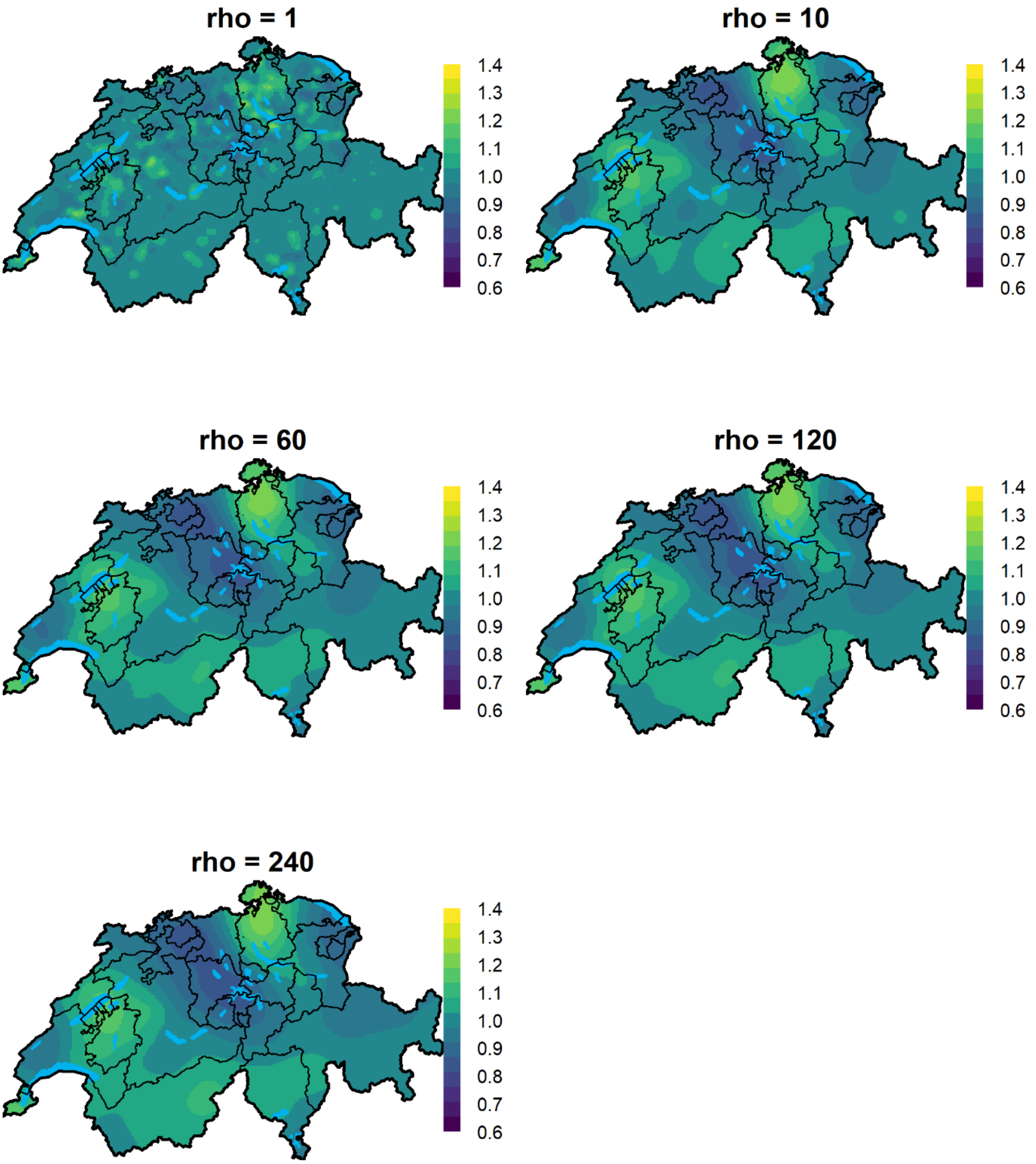

Figure S22. Boxplots of relative risk surfaces of CNS tumours using log-Gaussian Cox processes, the unadjusted model and different priors for the range parameter: Boxplots of the median posterior of grid specific relative risk using different penalized complexity (PC) priors for the range parameter (ρ) and focusing on CNS tumours and place of diagnosis using the unadjusted model. Notice that for the model presented in the paper we used $\rho = 60m$.

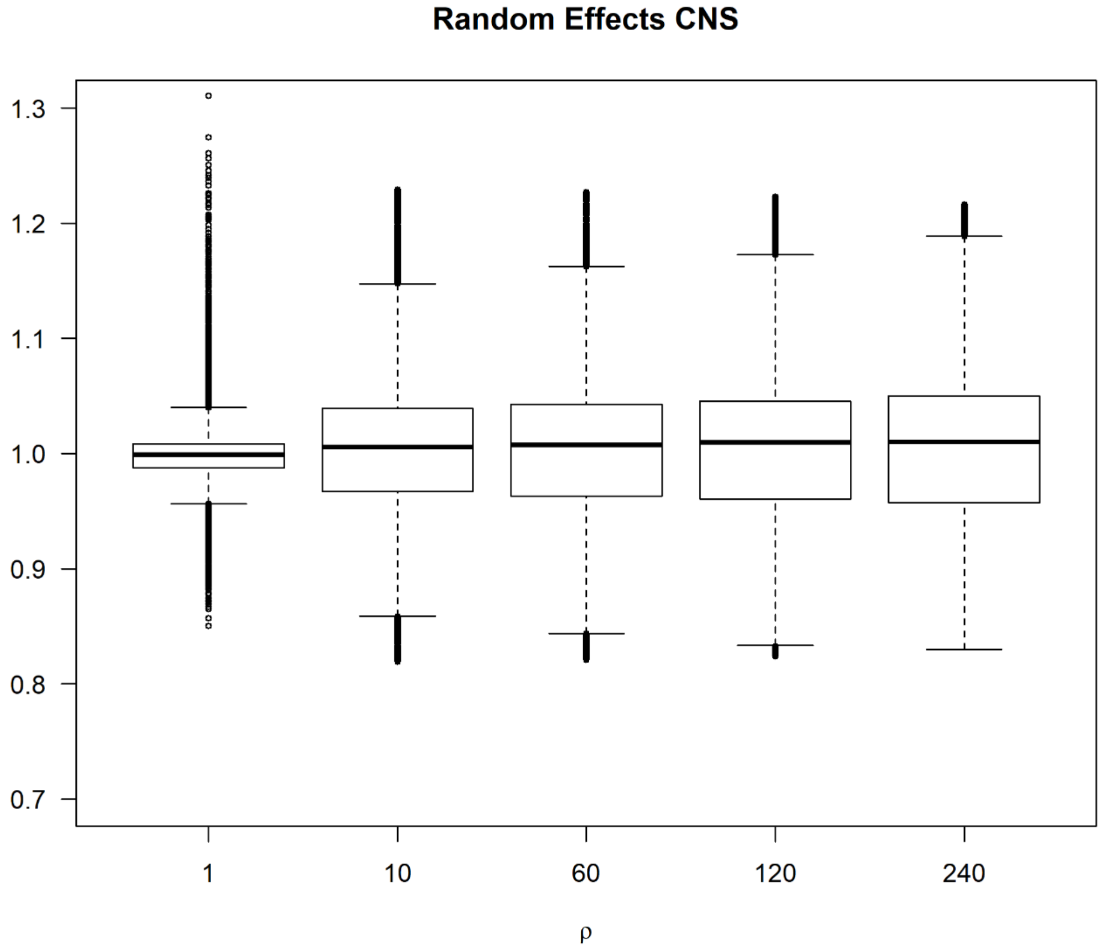

Figure S23. Sensitivity of relative risk surfaces of CNS tumours using log-Gaussian Cox processes, the fully adjusted model and different priors for the range parameter: Sensitivity analysis of the median posterior of grid specific relative risk using different penalized complexity (PC) priors for the range parameter (ρ) and focusing on CNS tumours and place of diagnosis using the fully adjusted model (adjusted for modelled ambient NO_2, modelled exposure to background ionising radiation, duration in years of general cancer registration in the canton, linguistic region and degree of urbanicity). Notice that for the model presented in the paper we used $\rho = 60m$.

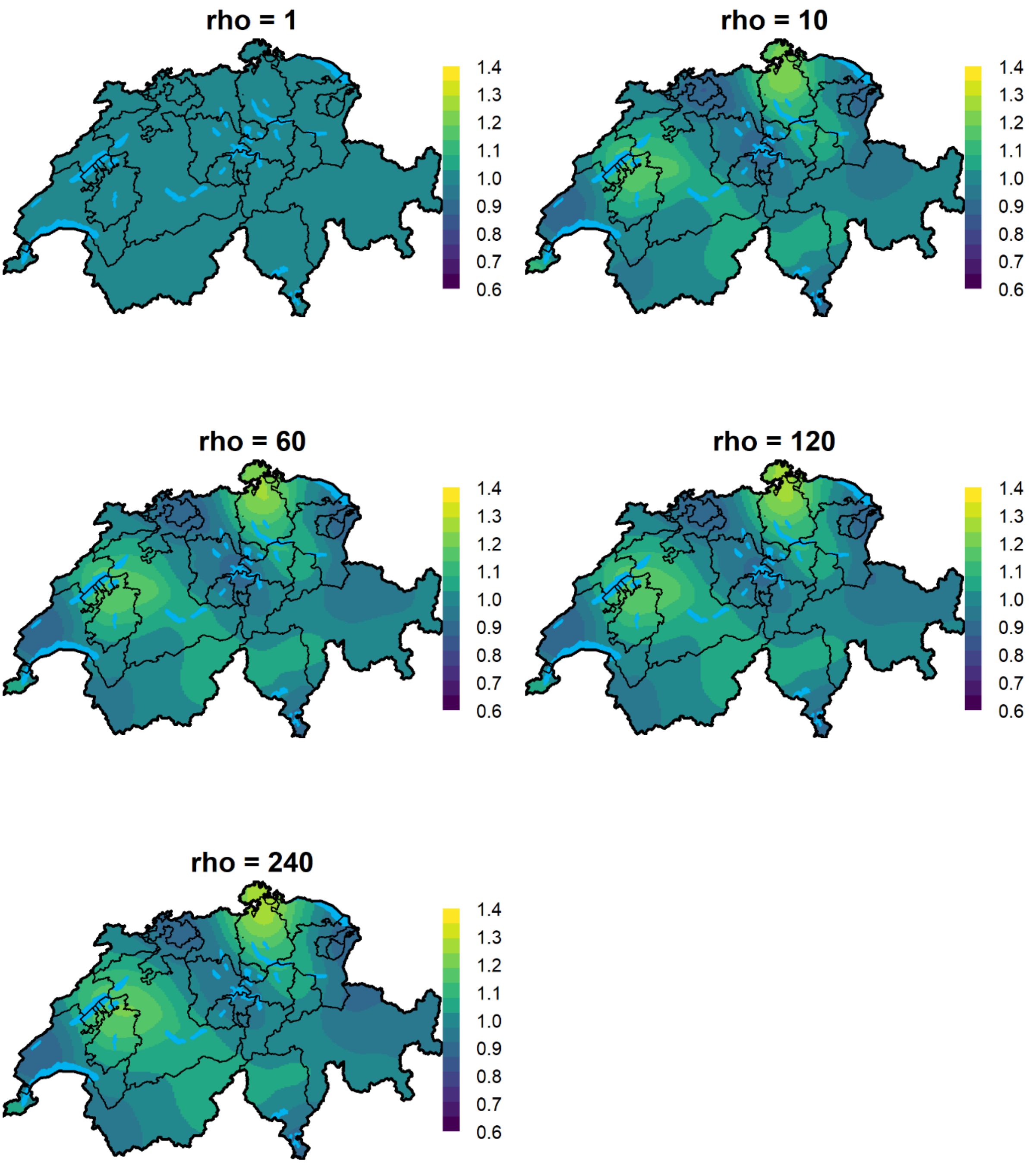

Figure S24. Boxplots of relative risk surfaces of CNS tumours using log-Gaussian Cox processes, the fully adjusted model and different priors for the range parameter: Boxplots of the median posterior of grid specific relative risk using different penalized complexity (PC) priors for the range parameter (ρ) and focusing on CNS tumours and place of diagnosis using the fully adjusted model (adjusted for modelled ambient NO_2, modelled exposure to background ionising radiation, duration in years of general cancer registration in the canton, linguistic region and degree of urbanicity). Notice that for the model presented in the paper we used $\rho = 60m$.

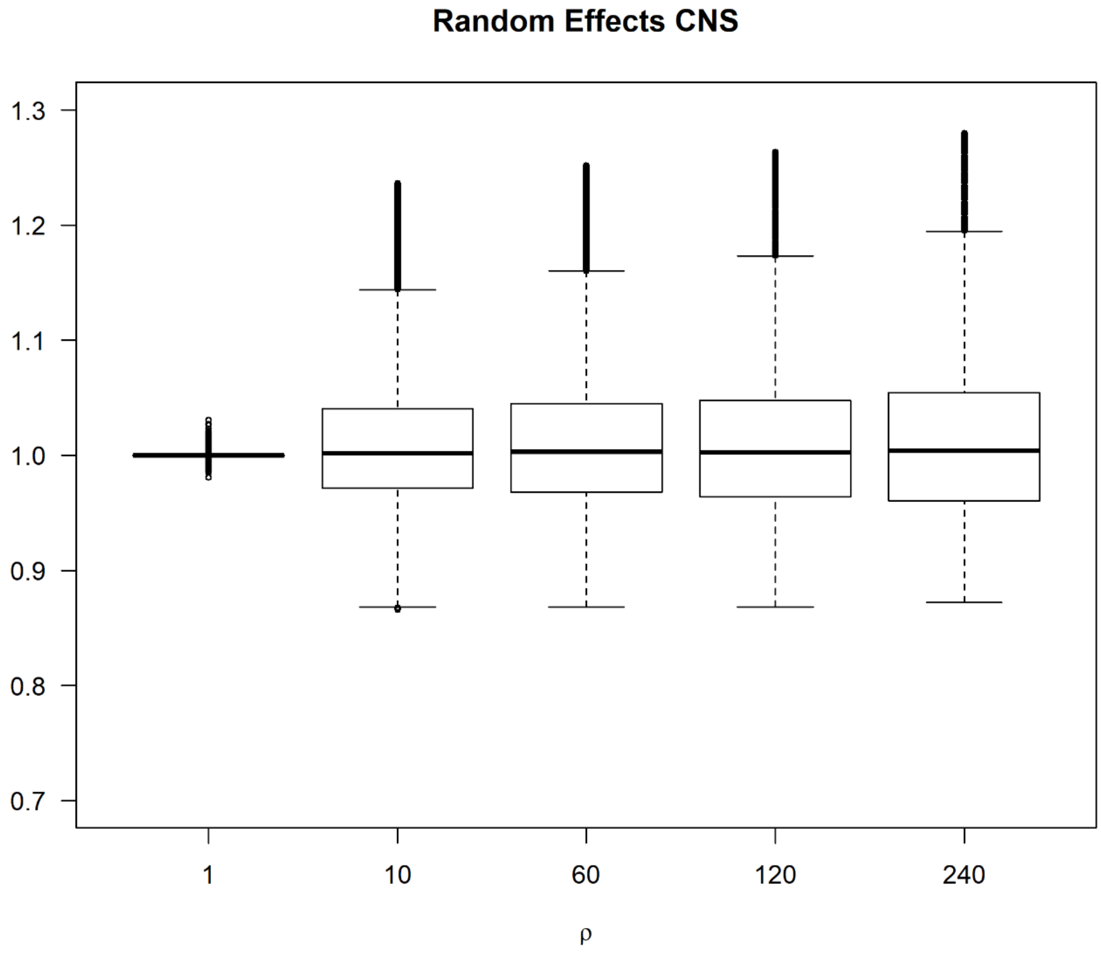

Figure S25. Regression coefficients of CNS tumours using log-Gaussian Cox processes, the fully adjusted model and different priors for the range parameter: Sensitivity analysis of the posterior distribution of the regression coefficients using different penalized complexity (PC) priors for the range parameter (ρ) and focusing on CNS tumours and place of diagnosis using the fully adjusted model (adjusted for modelled ambient NO_2, modelled exposure to background ionising radiation, duration in years of general cancer registration in the canton, linguistic region and degree of urbanicity). Notice that for the model presented in the paper we used $\rho = 60m$.

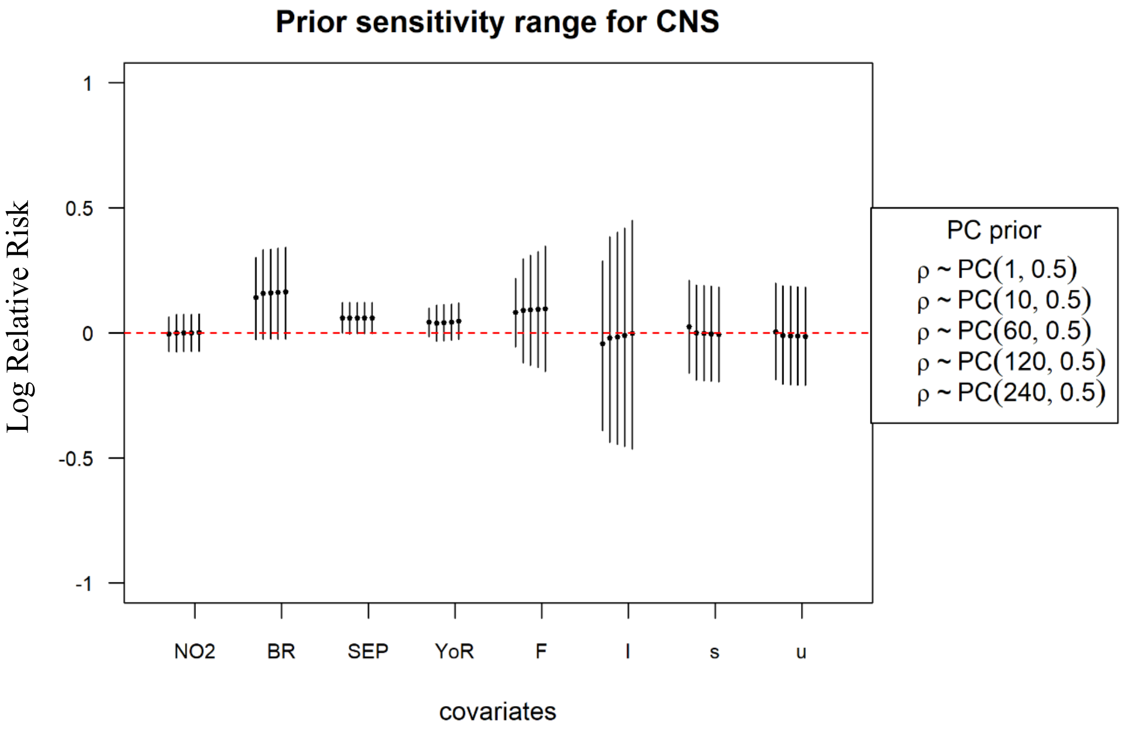

Abrevations: NO_2: Nitrogen Dioxide (Figure S3), BR: Total dose background radiation (Figure S4), SEP: Socio-Economic Position (Figure S5), YoR: years of existing cantonal registry (Figure S6), F: French speaking part, I: Italian speaking part (Figure S7), s: semi-urban areas, u: urban areas (Figure S8)

NO_2, background ionising radiation, SEP and years of cantonal registry were scaled and considered as linear effects. Their interpretation is a mulitplicative increase (or dicrease) in the number of observed cases compared to the number of the expected cases per 1sd increase (or dicrease) in the covariate. The sd for NO_2 is 77.7 $\mu g/m^3 \times 10$, for total background radiation 60.2 nSv/h, for SEP 8.7 units and for years of cantonal registry 11.6 years.

Figure S26. Post-hoc analysis for CNS tumours restricting to cases (n=968) diagnosed during 1995-2015. Median posterior of grid specific relative risk and exceedance probability were calculated based on a log-Gaussian Cox process model at residence of diagnosis.

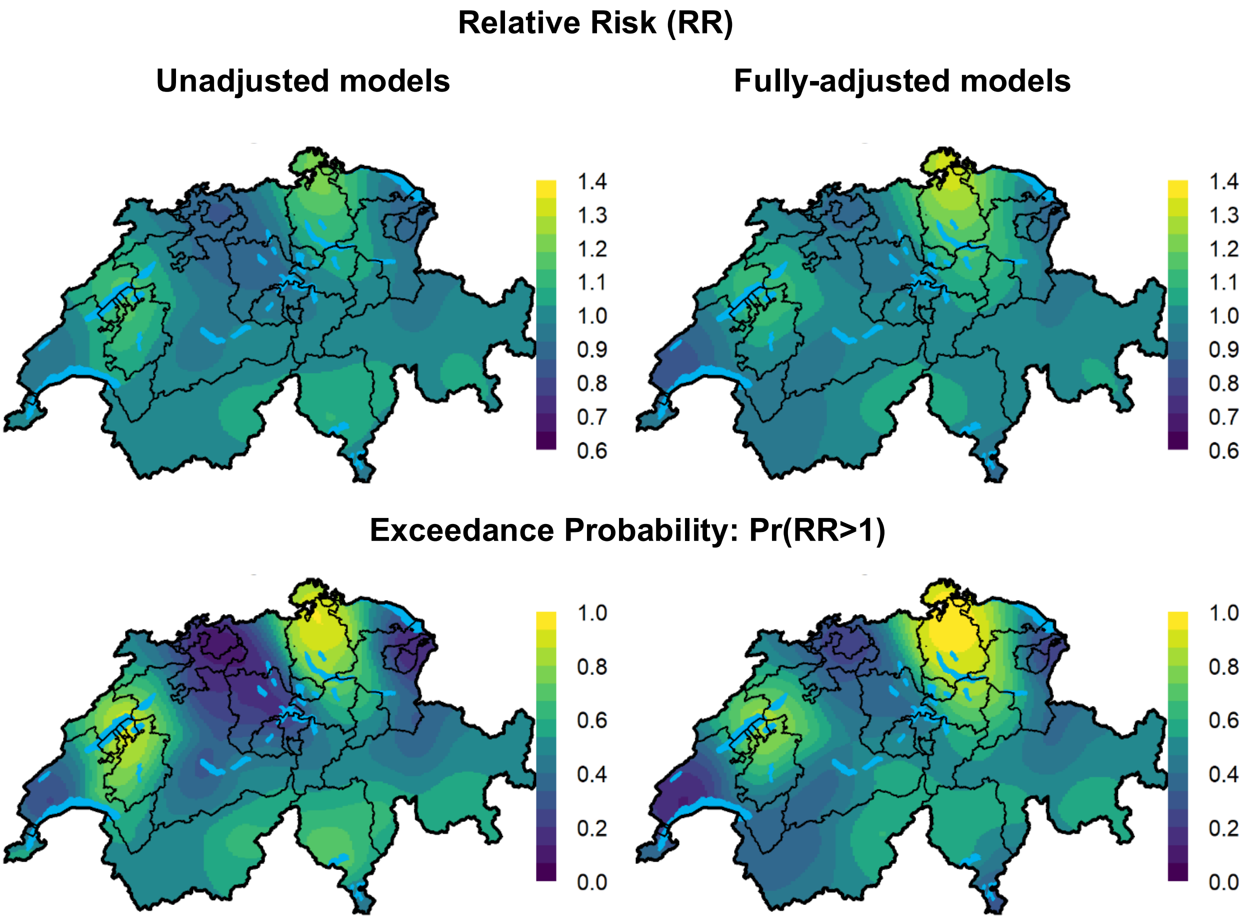

Unadjusted model: model without covariates, adjusted for age and year of diagnosis through the indirect standardisation

Adjusted model: additionally adjusted for modelled ambient NO_2 air concentration (Figure S3), modelled exposure to background ionising radiation (Figure S4), Swiss socioeconomic index (Figure S5), duration in years of general cancer registration in the canton (Figure S6), linguistic region (Figure S7) and degree of urbanicity (Figure S8) through inclusion of spatial covariates.

Figure S27. Relative risk surfaces of subgroups of CNS tumours: Post-hoc analysis showing the median posterior of grid specific relative risk of childhood CNS tumours focusing on the three main diagnostic subgroups, namely astrocytoma (n = 511), embryonal CNS (n=266) and other CNS tumours (n = 512). Results were calculated based on a log-Gaussian Cox process model at residence of diagnosis. Notice that the limits of the colourkeys are not the same as the ones used to report the rest of the maps in the paper (here they range from 0.6 to 1.6, whereas for the rest of the maps from 0.6 to 1.4).

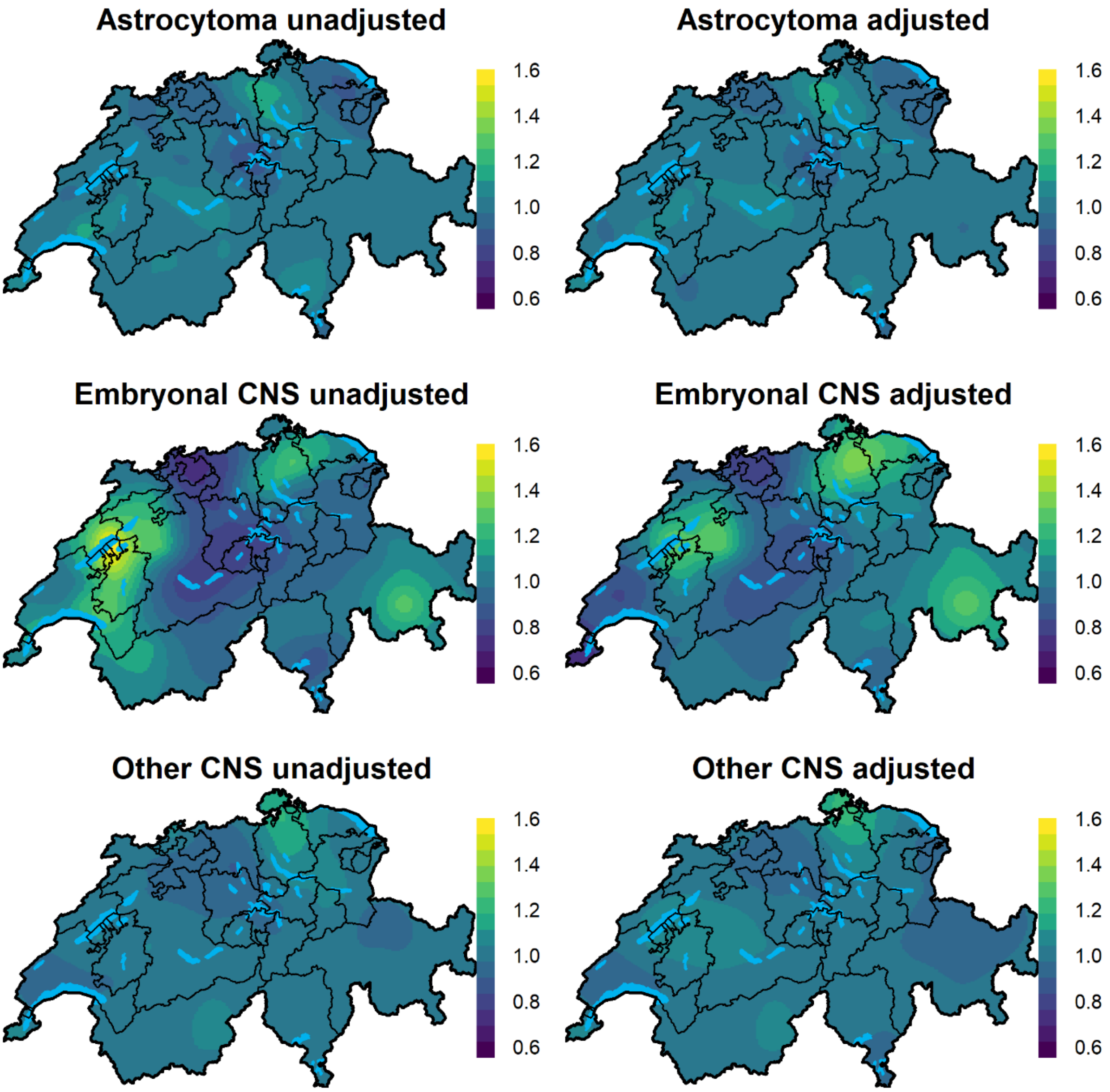

Unadjusted model: model without covariates, but adjusted age and year of diagnosis through the offset

Adjusted model: additionally adjusted for modelled ambient NO_2 air concentration (Figure S3), modelled exposure to background ionising radiation (Figure S4), Swiss socioeconomic index (Figure S5), duration in years of general cancer registration in the canton (Figure S6), linguistic region (Figure S7) and degree of urbanicity (Figure S8) through inclusion of spatial covariates.

Figure S28. Exceedance probability surfaces of subgroups of CNS tumours: Post-hoc analysis showing the exceedance probability, i.e. Pr(RR >1), where RR is the posterior of the grid specific relative risk of childhood CNS tumours focusing on the three main diagnostic subgroups, namely astrocytoma (n = 511), embryonal CNS (n=266) and other CNS tumours (n = 512). Results were calculated based on a log-Gaussian Cox process model at residence of diagnosis.

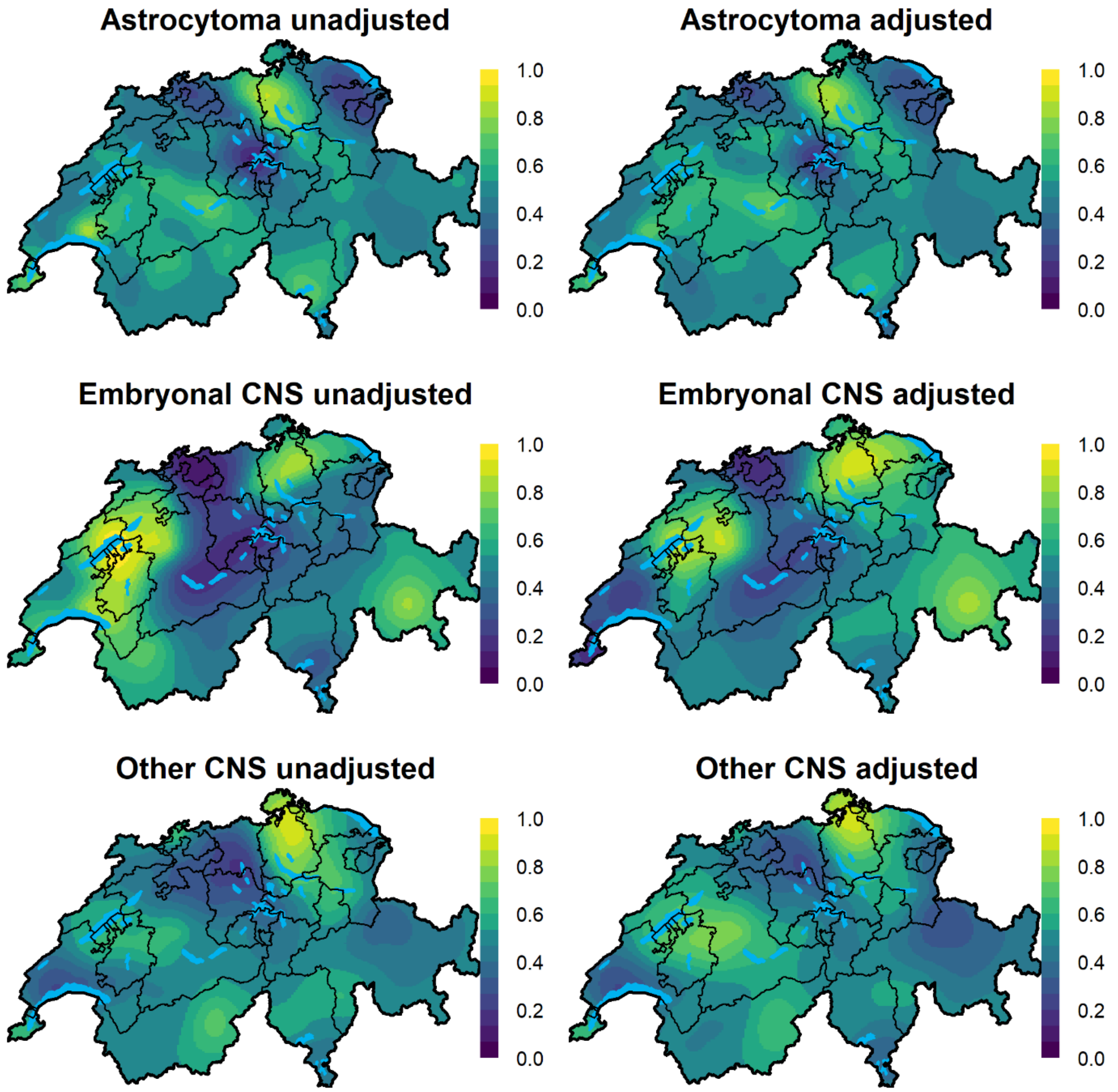

Unadjusted model: model without covariates, but adjusted for age and year of diagnosis through the offset

Adjusted model: additionally adjusted for modelled ambient NO_2 air concentration (Figure S3), modelled exposure to background ionising radiation (Figure S4), Swiss socioeconomic index (Figure S5), duration in years of general cancer registration in the canton (Figure S6), linguistic region (Figure S7) and degree of urbanicity (Figure S8) through inclusion of spatial covariates.

References

1. Besag J, York J, Mollié A: A Bayesian image restoration with two applications in spatial statistics. Ann Inst Statist Math 1991, 43:1–59.
2. Sorbye SH, Rue H: Scaling intrinsic Gaussian Markov random field priors in spatial modelling. Spat Stat-Neth 2014, 8:39-51.
3. Freni-Sterrantino A, Ventrucci M, Rue H: A note on intrinsic conditional autoregressive models for disconnected graphs. Spatial and spatio-temporal epidemiology 2018, 26:25-34.
4. Riebler A, Sorbye SH, Simpson D, Rue H: An intuitive Bayesian spatial model for disease mapping that accounts for scaling. Statistical methods in medical research 2016, 25(4):1145-1165.
5. Simpson D, Rue H, Riebler A, Martins TG, Sørbye SH: Penalising model component complexity: A principled, practical approach to constructing priors. Statistical Science 2017, 32(1):1-28.
6. Rybach L, Bachler D, Bucher B, Schwarz G: Radiation doses of Swiss population from external sources. J Environ Radioact 2002, 62(3):277-286.
7. Rybach L, Schwarz GF, Medici F: Construction of radioelement and dose rate baseline maps by combining ground and airborne radiometric data. In: Uranium exploration data and techniques applied to the preparation of radioelement maps, Proceedings of a Technical Committee meeting held in Vienna, 13-17 May 1996. Volume IAEA-Tecdoc-980, edn. Vienna, Austria: International Atomic Energy Agency (IAEA); 1996: p. 33-44.
8. Panczak R, Galobardes B, Voorpostel M, Spoerri A, Zwahlen M, Egger M, Swiss National C, Swiss Household P: A Swiss neighbourhood index of socioeconomic position: development and association with mortality. Journal of epidemiology and community health 2012, 66(12):1129-1136.
